# Altered microheterogeneity at several N‐glycosylation sites in OPSCC in constant protein expression conditions

**DOI:** 10.1096/fba.2023-00066

**Published:** 2023-12-14

**Authors:** Amy Dickinson, Sakari Joenväärä, Tiialotta Tohmola, Jutta Renkonen, Petri Mattila, Timo Carpén, Antti Mäkitie, Suvi Silén

**Affiliations:** ^1^ Department of Otorhinolaryngology—Head and Neck Surgery University of Helsinki and Helsinki University Hospital Helsinki Finland; ^2^ Research Program in Systems Oncology, Faculty of Medicine University of Helsinki Helsinki Finland; ^3^ Transplantation Laboratory, Haartman Institute University of Helsinki Finland; ^4^ HUSLAB Helsinki University Hospital Helsinki Finland; ^5^ Department of Pathology University of Helsinki and HUS Helsinki University Hospital Helsinki Finland; ^6^ Division of Ear, Nose and Throat Diseases, Department of Clinical Sciences, Intervention and Technology Karolinska Institutet and Karolinska Hospital Stockholm Sweden

**Keywords:** alpha‐1‐antitrypsin, glycosylation, IgA, N‐glycopeptides, oropharyngeal squamous cell carcinoma

## Abstract

Protein glycosylation responds sensitively to disease states. It is implicated in every hallmark of cancer and has recently started to be considered as a hallmark itself. Changes in N‐glycosylation microheterogeneity are more dramatic than those of protein expression due to the non‐template nature of protein glycosylation. This enables their potential use in serum‐based diagnostics. Here, we perform glycopeptidomics on serum from patients with oropharyngeal squamous cell carcinoma (OPSCC), compared to controls and comparing between cancers based on etiology (human papilloma virus‐ positive or negative). Using MS2, we then targeted glycoforms, significantly different between the groups, to identify their glycopeptide compositions. Simultaneously we investigate the same serum proteins, comparing whether N‐glycosylation changes reflect protein‐level changes. Significant glycoforms were identified from proteins such as alpha‐1‐antitrypsin (SERPINA1), haptoglobin, and different immunoglobulins. SERPINA1 had glycovariance at 2 N‐glycosylation sites, that were up to 35 times more abundant in even early‐stage OPSCCs, despite minimal differences between SERPINA1 protein levels between groups. Some identified glycoforms' fold changes (FCs) were in line with serum protein level FCs, others were less abundant in early‐stage cancers but with great variance in higher‐stage cancers, such as on immunoglobulin heavy constant gamma 2, despite no change in protein levels. Such findings indicate that glycovariant analysis might be more beneficial than proteomic analysis, which is yet to be fruitful in the search for biomarkers. Highly sensitive glycopeptide changes could potentially be used in the future for cancer screening. Additionally, characterizing the glycopeptide changes in OPSCC is valuable in the search for potential therapeutic targets.

## Graphical abstract

Oropharyngeal squamous cell carcinoma (OPSCC) and control serum samples were processed, isolating then digesting proteins, then enriching into glycopeptides and peptides (1–3). After proteomics, glycopeptides were quantified with MS(4A). Glycopeptide expressions were compared between groups (5), then significantly different glycopeptides targeted using MS^2^ for identification(4B). A representative alpha‐1‐antitrypsin glycopeptide (4), almost absent in controls but in significantly higher levels in OPSCC (representing, e.g., a fold change of 35 between control and early‐stage HPV+), despite minimal protein‐level differences. Identified significant glycovariants in OPSCC are shown in the article.

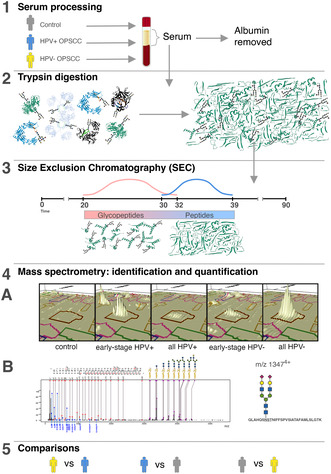

**Abbreviations**
ACN: acetonitrile
FA: formic acid
FC: fold change
HexNAc: N‐acetylhexosamine
HP: haptoglobin
HPV: human papillomavirus
IGHA1: immunoglobulin heavy constant alpha 1
IGHA2: immunoglobulin heavy constant alpha 2
IGHG2: immunoglobulin heavy constant gamma 2
MS: mass spectrometry
OPSCC: oropharyngeal squamous cell carcinoma
PCA: principal component analysis
SERPINA1: alpha‐1‐antitrypsin

## 1 INTRODUCTION

Protein glycosylation is an important non‐template post‐translational modification, critical to homeostasis. Not only affecting the physical properties of the protein, such as the stability, folding, solubility of the protein,^1^ protein glycosylation contributes to protein secretion and transport, the immune response and epitopes, and receptor interactions,^2^ among other things. Glycosylation is a tightly controlled process, remarkably stable in an individual until their homeostasis is affected, be it from disease or lifestyle change.^3^ Small changes in environmental clues, as in pathological states, can alter the activity of the enzymes and glycosylation occurring in the cells.^4^, ^5^

Protein glycosylation begins in the endoplasmic reticulum (ER), in a process general to all cells, where a pre‐formed glycan is transferred from a lipid carrier molecule to a nascent protein at a glycosylation site. In N‐glycosylation, the glycosylation site consists of the following amino acid consensus sequence: either Asparagine—X—serine/threonine (rarely cystine), where X is any amino acid except proline. There may be one or multiple potential N‐glycosylation sites on any given glycoprotein, giving rise to different glycovariants. The glycan is then progressively trimmed of monosaccharide molecules in the ER, then the Golgi, whereby progressive monosaccharide molecules are attached to or removed from the glycan in question by the action of highly specific enzymes in the ER and different areas of the Golgi.^6^ The enzymes can add new antennae to the glycan, can bisect other structures on the glycan, and can add monosaccharides such as fucose, or sialic acid to the core or antennae of the glycan. The glycan‐modifications performed in the Golgi are tissue‐ and physiological‐state specific. Different cells have different expressions of types of glycosylation enzymes, which also may change according to the cell cycle.^1^ Glycoproteins are released from the Golgi through highly regulated processes via vesicle trafficking to their final location, for example the bloodstream, where they can be detected.

Glycans play a role in every recognized cancer hallmark.^7^ As cancer is a process by which the cells that evade destruction survive and thrive, likely the changes were selected as they convey an evolutionary advance to the cancer cells.^8^ On the other hand, such modifications by the immune system may indicate a change in state in response to a changing immune state in cancer.^4^ Altered glycan expression can be attributed to transcription‐level dysfunction of glycosyltransferases, chaperone function dysregulation, altered glycosidase activity, altered expression of relevant glycosyltransferases in the Golgi, peptide backbone alterations, and availability of sugar nucleotide donors and cofactors.^9^

Certain glycan alterations commonly appear in cancer, some correlated with malignant transformation and cancer progression (e.g., sialylation or core fucosylation). Sialylated carbohydrates play a key role in cellular recognition, cell signaling, and cell adhesion.^9^ For the early detection of hepatocellular cancer, core fucosylation of alpha‐fetoprotein is an approved biomarker, differentiating hepatocellular carcinoma from cirrhosis and chronic hepatitis, thus improving the specificity of alpha‐fetoprotein as a biomarker.^9^, ^10^ Prostate specific antigen, a clinically used biomarker for prostate cancer, has been shown to be more specific for cancer when looking at its glycosylation profiles.^11^ Not only is the detection of cancers important, also is reducing false‐positives and the unnecessary distress, risks and costs associated with unnecessary investigations. This is an area glycosylation profiling could address.

Oropharyngeal squamous cell carcinomas (OPSCCs) are caused by smoking, alcohol, and/or human papillomavirus (HPV). HPV+ and HPV− tumors exhibit different behaviors, such as HPV+ OPSCCs having better prognosis despite metastasizing to regional lymph nodes at an early stage. They have thus been recently classified as separate entities by the Union of International Cancer Control, with different staging systems, using the surrogate tissue biomarker p16 to differentiate between the two.^12^

OPSCC's annual incidence is approximately 140,000.^13^ A recent dramatic rise of HPV‐related OPSCCs in developed nations has raised concerns of an OPSCC cancer epidemic.^14^ Diagnosis currently requires a biopsy from a clinically visible tumor, usually from a symptomatic patient^15^ as no serum biomarkers exist in clinical practice.^16^ In general, the earlier the OPSCC is detected, the less extensive the treatment required^17^ and the better the survival.^18^ Treatment options for OPSCC are surgery, radio‐, or chemotherapy or a combination of these.^17^ The full biological nature of OPSCC has not yet been elucidated in enough detail to be able to provide or even investigate any novel design treatments targeted to specific molecular structures, for example glycan structures or their targets.^19^

Traditional glycoprotein research has aimed at identifying global glycan alterations by cleaving glycans from the peptide chain, then analyzing the free glycans; however important glycosylation‐site information is lost. Recent focuses in the glycoproteomic community have been detecting intact N‐glycopeptides. The type of glycan and its glycosylation site can drastically change the behavior and characteristics of the protein^4^; this glycan/glycosylation site information may provide valuable insights and avenues for further study. Our group has previously developed a workflow for quantitative N‐glycopeptidomics, that both quantify N‐glycopeptides, and identifies their peptide sequence, proposing the glycoform's composition.^20^, ^21^, ^22^, ^23^

Current understanding of the biological significance of individual changes in glycosylation is still very limited, but sensitive reliable changes could be taken advantage of in diagnostics, prognostics, and therapeutics of disease. The main purpose of this study is thus to identify and quantify significant serum glycopeptides in OPSCC compared to controls. In order to understand whether changes in N‐glycosylation were coupled to the protein expression change (i.e., resulting from protein expression changes), we simultaneously performed proteomics of the same samples, comparing fold changes (FCs) between protein expression and glycopeptide expression. We used serum from 74 OPSCC (8 early‐stage HPV−, 24 early‐stage HPV+, 26 all‐stage HPV−, 48 all‐stage HPV+) and 12 healthy controls.

## 2 MATERIALS AND METHODS

### 2.1 Workflow

We used a case–control study design to compare (a) serum proteins and glycopeptides of healthy controls with patients with OPSCC, and (b) between patients with HPV‐driven OPSCC with non‐HPV‐driven OPSCC. We performed comparisons between OPSCC and controls, stratified by HPV, and by early‐ and late cancer stages. Additionally, we compared serum glycopeptides and proteins from serum of patients OPSCCs according to the HPV status (HPV+ vs. HPV−).

**FIGURE 1:**
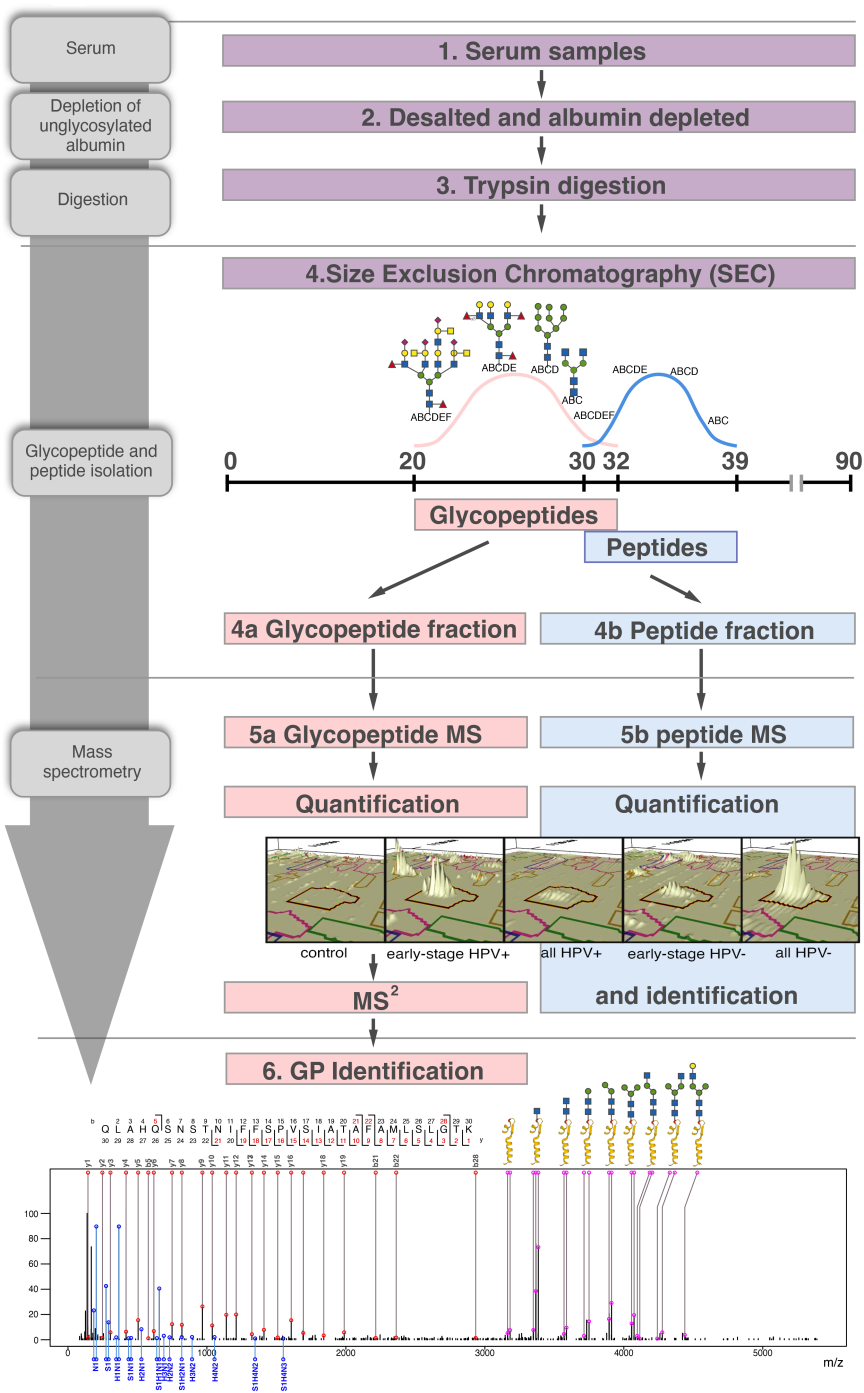
Workflow. General workflow. (1–3) after preparing and trypsin digesting the samples, we (4) separated them into glycopeptide‐ and peptide‐ enriched fractions using size exclusion chromatography, in which samples are separated from largest to smallest sizes. (5) mass spectrometry (MS) was performed on the peptide‐ and glycopeptide‐enriched samples. Here, we show an example 3‐D peak of the glycoform 1 (m/z 1347 m/z) ion in the different groups. To get these peaks, the mass spectrometer records the liquid chromatography retention time of the ions and the m/z (mass: charge ratio). The mass spectrometer oscillates between low‐ and elevated energy. At low energy, intact peptide ion m/z are recorded. At elevated energy, the peptide fragments are recorded. As the peptide fragments' retention time is the same as the intact peptide, the otherwise independent peptide fragments can be mapped to the intact peptide. Proteins are quantified and identified, and the glycopeptides are quantified based on this first MS step. The ion peak shown is delineated by the brown outline (and the surrounding, colored boxes are other ions). The contents of the delineated area are used to quantify the abundance of the ion within. All such ions are quantified and compared between groups, and an MS^2^ round, is performed on the significantly different glycopeptides to identify them based on their fragmentation pattern. (6) Example deconvoluted glycopeptide spectrum from the glycoform 1, showing the fragmentation from MS^2^. The peptide ion fragment peaks can be seen with red circles, corresponding to a peptide chain fragment at various b/y sites. The glycopeptide fragment peaks can be seen on the right with pink circles, indicating an intact peptide with the attached glycan shown diagrammatically. The blue circles indicate the glycan fragments.

Figure 1 shows the workflow. First, the serum samples^1^ from patients with OPSCC and control samples were processed by removing the most abundant and non‐glycosylated protein, albumin,^2^ then digesting the remaining proteins with trypsin.^3^ After this, we used size exclusion chromatography^4^ to separate the serum samples into fractions containing glycopeptides (4a) and fractions containing peptides (4b). We quantified the glycopeptides from the glycopeptide fraction using an MS protocol (5a), as our group has previously performed.^21^, ^22^, ^23^, ^24^ Following this, we used a targeted approach to identify the compositions of glycopeptides of interest, that is, those that were significantly different between our groups of interest: Statistically significant glycopeptide ions in any comparison were targeted in MS^2^ to determine the amino acid sequence, glycan composition, and proposed glycan structure for the ion.^6^ In parallel with the glycopeptide analysis, we also quantified and identified the proteins from the peptides fractions from the same patients/controls using an MS approach (5b), as our group have previously performed^25^, ^26^, ^27^, ^28^ except without removing the top 12 proteins. Here, we only removed albumin, which is not glycosylated, so that the proteins and glycopeptides could be directly comparable.

### 2.2 Patients and sera samples

Pre‐treatment serum samples were collected from patients diagnosed with non‐metastatic OPSCC between the years 2012 and 2015 at the Department of Otorhinolaryngology—Head and Neck Surgery, Helsinki University Hospital, Helsinki, Finland. Exclusion criteria were presence of coexisting inflammatory diseases, concurrent cancers, history of previous cancer within 18 years, and/or subsequent second primary cancer. Serum of 12 control patients were received from the Finnish Red Cross Blood Service. After collection, samples were allowed to clot at room temperature (RT) before being centrifuged at 4°C (1000×*g*) to separate serum. Sera were stored at −72°C until all were assayed at the same time. All experiments were performed in accordance with relevant guidelines and regulations.

Clinical data were collected from patient records. The UICC (Union for International Cancer Control) 8th edition TNM classification^29^ was used to evaluate tumor stage. In this paper, we used p16 as a surrogate marker for HPV. The groups were as follows: stage I HPV+ OPSCC (*n* = 24); stage I‐II HPV− OPSCC (*N* = 8); all HPV+ OPSCC (*n* = 48); all HPV− OPSCC (*n* = 26). Clinical data are shown in Table S1.

Written informed consent was obtained from all patients. The study plan was approved by the institutional Research Ethics Board at the Helsinki University Hospital (DNr. 51/13/03/02/2013). The study was performed in accordance with the Declaration of Helsinki.

### 2.3 Serum processing and protein digestion

Serum samples were desalted using Zeba™ Spin desalting plates (Thermo Scientific, MA, USA) prior to 50 μL aliquots being depleted of excess albumin using the Pierce™ albumin depletion kit (ThermoFisher Scientific, MA, USA). The remaining protein concentration was determined using the Bradford MX Protein Assay (Expedeon, Germany), after which the samples were dried then resuspended with 35 μL 6 M urea, then as previously described by Kontro et al., reduced, then alkylated prior to trypsin digestion.^24^ The samples were diluted 1:10 with 50 mM Tris solution to reduce the urea concentration; then trypsin (from bovine pancreas [Sigma Aldrich, MO, USA]) was added at a mass ratio of 1:50 and incubated at 37°C overnight. These samples were then dried and frozen at −72°C until further use.

### 2.4 Size exclusion chromatography

As previously described by our group, we performed size exclusion chromatography.^22^ Dried trypsinized samples were resuspended in 55 μL 0.1% formic acid (FA) (LiChropur® FA 98%–100% for HPLC (Merck, Germany)), centrifuged to pellet any precipitated molecules, then 50 μL of each supernatant was loaded into the size exclusion column (GE Healthcare Superdex™ peptide 3.2/300), and was run at an isocratic gradient of 0.05 mL/min of 0.1% FA. In order to determine the correct fractions, we originally collected fractions at 1‐min intervals from a pooled serum sample from 17 to 42 min, after which we screened each 1‐min fraction using UPLC‐MS^E^. We detected glycopeptides from 20 to 32 min and peptides from 30 to 39 min. We then performed size exclusion chromatography on 50 μL of each digest, collecting from each serum sample 2 aliquots: one containing glycopeptides and one containing peptides, which then were dried.

The glycopeptide samples were made up to a final concentration of 0.1% FA, 2.5% acetonitrile (ACN) in water, guided by measured protein concentrations, before being transferred to total recovery Waters mass spectrometry (MS) vials. 1uL peptide fractions, equivalent to ∼500 ng of total protein, were made up to 0.1% FA, 5% ACN in water, and spiked with 50 fmol per injection *E. coli* Hi3 peptide standard in UPLC‐grade water, also according to peptide concentration (Pierce Quantitative Fluorometric Peptide Assay Kit (Pierce Quantitative Fluorometric Peptide Assay Kit (ThermoFisher Scientific, MA, USA).

Peptides and glycopeptides were then run on a mass spectrometer using parameters optimized for peptides and glycopeptides, respectively, described below.

### 2.5 Mass spectrometry

The nanoAcquity UPLC system (Waters Corporation, MA), joined to the Synapt G2‐Si HDMS (Waters Corporation, MA), was used, and the TRIZAIC nanotile (Waters Corporation, MA) was used as separating device prior to MS of both N‐glycopeptide‐ and peptide‐enriched fractions. Calibration was performed with sodium iodide clusters over mass range of 50–2500 m/z. The whole study was done in the positive mode. Leucine enkephalin (C25H37O7, M+H+ 556.2771 m/z, 1 ng/μL in 50:50 ACN: water +0.1% FA) was infused into the reference sprayer 300 nL/min to act as a lock mass for MS data analysis.

Samples were kept at −20°C until being placed in the sample manager at 4°C for up to 24 h. UPLC was used for all sample acquisitions. For liquid chromatography, we used Buffer A: (0.1% FA in UPLC‐grade water) and Buffer B (0.1% FA in ACN). UPLC analytical gradients are shown in the Table 1.

**TABLE 1:** UPLC gradients used for the (A) proteomics and (B) N‐glycopeptide runs, showing also the gradients used for trapping prior to the runs. Buffer A is 0.1% FA in UPLC‐grade water. Buffer B is 0.1% FA in ACN.

Time (min)	Flow rate (μL/min)	Buffer A %	Buffer B %
(A) Proteomics, trapping			
0	4	97.5	2.5
4	4	97.5	2.5
Proteomics, runs			
0	0.45	2.5	97.5
1	0.45	1	99
5	0.45	5	95
65	0.45	30	70
78	0.45	50	50
80	0.45	85	15
85	0.45	85	15
86	0.45	2.5	97.5
90	0.45	2.5	97.5
100	0.45	2.5	97.5
(B) N‐glycopeptide quantification, trapping			
0	0.5	97.5	2.5
1	0.5	97.5	2.5
2	1.5	97.5	2.5
3	2	97.5	2.5
8	2	97.5	2.5
N‐glycopeptide runs			
0	0.1	2.5	97.5
1	0.15	3	97
2	0.25	3	97
3	0.45	5	95
48	0.45	50	50
50	0.45	90	10
55	0.45	90	10
56	0.45	2.5	97.5
65	0.45	2.5	97.5

Abbreviations: ACN, acetonitrile; FA, formic acid.

Calibration was performed with sodium iodide clusters over mass range of 50–2500 m/z by infusing 2 μg/μL sodium iodide solution in 50 2‐propanol/water to the mass spectrometer.

### 2.6 N‐glycopeptidomics

For N‐glycopeptide quantification, we used sensitivity MS^E^ mode. The following parameters were used: acquisition time 0–60 min; positive polarity; 50–2500 Da range; scan time: 1 s; collision energy: ramp in trap from 14 to 44 V in function 2 (high energy). 10% of samples were run in triplicate.

For N‐glycopeptide identification, we used Fast DDA mode and standard collision induced fragmentation with argon. The technical parameters used were as follows: acquisition time: 2–60 min; polarity: positive; analyzer mode: sensitivity; 50–2500 Da range; when the intensity threshold of 5000 was reached for 1 s, the system switched to MS^2^; scan time in MS mode: 1 s; 2 MS^2^ were allowed at the same time; MS^2^ scan time: 1 s; MS^2^ continued for 60s or until the total ion count reached 70 million, whichever came first; when an MS^2^ target ion had been acquired, we excluded it as well as ions +/− 1500 mDa from it, from being reacquired within the following 60 s; we acquired only the ions that we were targeting, with a mass window of +/−1500 mDA, +/− 120 s from the targeted ion and its retention time; collision energy ramp used was 20–60 eV.

### 2.7 Proteomics

UDMS^E^ in resolution mode was used for protein quantitation and identification.

### 2.8 MS data processing and statistical analysis

Both peptide quantification/identification and glycopeptides quantification raw files were imported to Progenesis QI for proteomics (Nonlinear Dynamics, NC, USA). Post‐acquisition mass correction was done when raw data were imported to Progenesis with lock mass ion of M+H+ 556.2771 m/z.

For the proteomics project, peptides were aligned and normalized with Hi3 E.coli peptides (Waters, MA, USA).^30^ For peptide identification, ion account was used, against the Uniport human database, search parameters on the Protein Lynx Global Server (PLGS) v3.03 search engine are as follows: cleaved by trypsin; 2 missed cleavages allowed; maximum protein was 250 kDa; fixed modification: carbamidomethyl cystine; variable modification: oxidation methionine; automatic tolerance parameters for peptide and fragment tolerance; maximal FDR 1%; default parameters for ion matching requirements. Quantification using non‐conflicting peptides was applied. The parsimony principle was used for protein grouping in the case of shared peptides. Only proteins with 2 or more unique peptides were included.

For glycopeptide quantification project, we performed manual curation of the glycopeptides ions that were selected for fragmentation with MS^2^. We included ions with charge states of 3–6 that were identified between 17 and 47 min of the MS run, and with an intensity above 10^3. We manually assessed each ion for quality of quantitation and suitability for fragmentation with MS^2^. We assessed the following aspects: the isotopic distribution of the ion, the chromatogram for the retention time to ensure that quantitation was accurately assessed; the surrounding of the selected ion were inspected for overlapping ions to avoid mixed ion spectra in MS^2^. The data were normalized to all potential glycopeptide ions present.

Principal component analyses (PCAs) were created for each comparison using Progenesis QI for Proteomics on the proteins (Figure S3) and on the potential glycopeptides ions identified (Figure S1.

To identify significantly different glycopeptides ions between the groups, the unpaired t‐test was used for parametric data and for non‐parametric data, the Mann–Whitney *U*‐test was used. The Benjamini–Hochberg procedure was performed as a multiple‐testing correction, with a cutoff of 0.05.

Only glycopeptides ions with a *p*‐value < 0.05 in their comparison were targeted for MS^2^ fast DDA runs.

### 2.9 Glycopeptide identification

MS^2^ spectra were manually deconvoluted in Waters MassLynx 4.1 software using the MaxEnt3 module and saved as peak lists. Detected glycopeptides were then identified using the web‐based software GlycopeptideID (Applied Numerics), which was developed for automated CID MS^2^ spectrum analysis. Although this software has been revised from earlier versions, the principle of this method is explained in detail in two publications by Joenvääräa et al.^20^ and Peltoniemi et al.^31^ Briefly, each spectrum was searched against the UniProt tryptic peptide database of known serum proteins and given a peptide score. Next, calculating glycan mass based on total mass minus proposed peptide mass, possible glycan compositions are fitted into the total mass, and the resulting glycopeptides are fitted into the acquired spectra and scored to give a glycan score. The total score is the combination of the peptide score + glycan score. The results are ranked, and for each possible result, an annotated spectrum is shown along with the b/y ions from the spectrum that match the most likely peptide chain (Figure 1, bottom panel, Figure S2). The glycan compositions are also shown, with the following abbreviations of each monosaccharide as follows: H: hexose; N: hexosamine; S; sialic acid; F: fucose. The file we used of known human glycans is also in the Supplements. The ion settings were as follows: ion: H; precursor and fragment tolerances: 35 ppm. 450 or fewer fragments were taken into the analysis for glycopeptide identification. The human glycan database was downloaded from GlycoMod,^32^ and in Glycopeptide ID, we allowed a maximum of five glycosidic bond cleavages to occur to create the theoretical fragmentation fingerprint for each glycan. The human proteome was downloaded from Uniprot and further in silica trypsin digested using the Glycopeptide ID, allowing for 2 missed cleavages, and carbamidomethyl cystine and/or oxidation methionine modifications. Finally, only the peptides with N‐glycosylation consensus sequence were included in the search database.

For glycopeptide identification, the peptide settings were as follows: ion series b/y; limit matches—output only peptides that pair with glycans, max charge of 4 for fragment matches; apply target decoy. FDR cutoff of 4.65 as a further filter for limiting glycopeptides matches. The glycan settings were as follows: de novo search allowed; peptide is not fragmented when attached to the glycan. The false‐discovery rate (FDR) is determined by searching the spectra against a reversed peptide database (target‐decoy search).

## 3 RESULTS/DISCUSSION

Using MS, we determined the relative abundances of plasma glycopeptides in patients with OPSCC and controls. We then targeted significantly different glycopeptides using an MS^2^ approach to determine the amino acid sequence, glycan composition, and proposed glycan structure for statistically significant glycopeptide ions. Using the same samples with the same pre‐processing, we performed in‐parallel proteomics, through which we were able to assess the serum protein expression changes of the protein backbones of the glycopeptides/glycoforms we elucidated. This way we can infer whether the glycosylation is coupled to the protein expression or independent.

In order to identify the glycoforms that become aberrant even at an early‐stage, that could be useful for early diagnostics, we compared the serum N‐glycopeptides in early‐stage HPV+ and HPV− patients with controls. PCAs of these ions separated patients with early‐stage tumors quite well from controls with minimal overlaps as would be expected from low‐grade tumors. By additionally comparing the serum N‐glycopeptides between late‐stage both HPV+ and HPV− OPSCCs with controls, we are able to assess whether the altered glycovariants follow the same pattern as the tumors progress.

After targeting for identification significantly different ions within groups, we identified 21 glycan variants from 4 plasma proteins, from known glycosylation sites. These glycoforms and abundances are shown in Table 2. Details of the MS data and proposed glycan structures are found in the Tables S2 and S3; Figures S2 and S4.

**TABLE 2:** Significant glycoforms. A glycoform is a specific peptide + glycan. Protein headers show the protein backbone from which glycoform was identified, using uniport database of known glycopeptides. Proteins are named with their name, their abbreviation, and their Uniprot unique protein identifier code. Each glycoform is identified with a number 1–21, and also with their m/z in the mass spectrometer and charge stage (z), for example, +4 or + 5. The N‐glycoprotein site from which the glycoform derives on the protein is shown as N‐site. The glycan composition is also described, S referring to sialic acid, H referring to hexose, N referring to HexNac, and F referring to fucose. The fold changed refer to the fold‐change differences at a group level, comparison groups shown on the top row. Those in bold are statistically significant after the multiple‐testing correction was applies. Those in black but not bold were statistically significant without multiple‐testing correction. Those in gray were not statistically significant. *p*‐values before multiple‐testing correction are shown on the right for each comparison/glycoform/protein. For example alpha‐1‐antitrypsin, uniprot code P01009, had 5 statistically significantly different glycoforms (1–5). Duplicate glycoforms (e.g., glycoform 1) refer to the different charge states the glycopeptide ion was identified as at the mass spectrometry quantification/identification levels.

Comparisons															
				Early HPV−/control	Non‐early HPV−/control	All HPV− /control	Early HPV+ /control	Non‐early HPV+ /control	All HPV+ /control	Early HPV−/control	Non‐early HPV−/control	All HPV− /control	Early HPV+ /control	Non‐early HPV+ /control	All HPV+/control
Glycoform #	m/z (z)	N_site	Composition	Fold changes						*p*‐values. Bold if also significant after Benjamini‐Hochberg procedure					
P01009		Alpha‐1‐antitrypsin (SERPINA1)		1.30	**1.82**	**1.63**	1.30	1.36	1.33	0.058	**0.001**	**0.005**	0.020	0.015	0.013
1	1078.06(5)	70	S2H5N4	**19.20**	**61.50**	48.50	**35.80**	37.50	38.80	**0.005**	**0.001**	0.004	**0.007**	0.050	0.017
1	1347.34(4)	70	S2H5N4	**10.30**	**27.20**	22.00	**14.20**	26.84	21.71	**0.000**	**0.003**	0.006	**0.001**	0.010	0.008
2	1384.10(4)	70	S1H5N4F3	10.40	58.50	43.70	19.60	25.60	24.09	0.034	0.038	0.072	0.031	0.233	0.150
3	1150.10(5)	271	S1H5N4F2	**4.00**	16.80	12.85	**5.20**	13.10	9.63	**0.006**	0.072	0.111	**0.000**	0.038	0.050
4	1273.33(4)	271	H4N3F2	17.60	132.92	97.40	**24.50**	102.90	68.13	0.059	0.072	0.122	**0.002**	0.088	0.135
5	1437.13(4)	271	S2H5N4	6.70	30.80	23.40	**6.80**	19.70	14.03	0.012	0.118	0.162	**0.000**	0.063	0.085
P00738		Haptoglobin (HP)		1.00	**2.40**	**1.94**	1.40	1.20	1.32	0.901	**0.000**	**0.012**	0.021	0.213	0.063
6	927.90(4)	241	S1H5N4	−1.10	1.29	1.09	1.50	1.27	1.24	0.757	0.122	0.604	0.049	0.387	0.319
7	1000.68(4)	241	S2H5N4	−1.10	**1.96**	1.64	**1.60**	1.40	1.51	0.594	**0.001**	0.030	**0.006**	0.179	0.048
7	1060.98(4)	241	S2H5N4	1.30	2.26	1.97	1.30	−1.33	−1.00	0.410	0.049	0.090	0.155	0.154	0.999
8	1115.19(3)	241	S1H4N3	−1.10	**2.00**	1.68	**1.70**	1.50	1.60	0.714	**0.001**	0.018	**0.002**	0.069	0.016
9	977.62(5)	184	S2H5N4	1.90	**4.57**	**3.75**	**3.20**	1.87	2.52	0.007	**0.000**	**0.000**	**0.000**	0.015	0.002
9	1628.70(3)	184	S2H5N4	2.20	7.53	5.90	**3.10**	5.56	4.52	0.013	0.005	0.016	**0.009**	0.026	0.028
P01859		Immunoglobulin heavy constant gamma 2 (IGHG2)		1.10	1.17	1.16	−1.10	1.11	1.02	0.486	0.338	0.308	0.511	0.081	0.911
10	868.01(3)	176	H3N4F1	−2.00	2.18	1.66	**−2.50**	4.53	2.66	0.035	0.233	0.437	**0.000**	0.030	0.229
11	852.86(4)	176	H5N4F1	**−2.10**	14.72	10.33	**−2.50**	28.60	15.70	**0.001**	0.087	0.173	**0.000**	0.023	0.130
12	922.03(3)	176	H4N4F1	−2.50	1.72	1.31	**−3.10**	4.20	2.44	0.020	0.384	0.658	**0.000**	0.014	0.197
12	812.34(4)	176	H4N4F1	−1.40	19.70	13.90	**−1.80**	45.50	25.00	0.118	0.061	0.134	**0.003**	0.024	0.127
P01876/P01877		Immunoglobulin heavy constant alpha 1/2 (IGHA1/2)		**1.5/1.5**	**1.96/1.81**	**1.8/1.7**	1.2/1.0	1.40/1.23	1.31/1.13	**0.004/ 0.002**	**0.003/0.007**	**0.003/0.005**	0.053/ 0.806	0.538/ 0.048	0.05/ 0.33
13	984.44(5)	144/131	S1H4N5	1.90	5.29	4.23	**2.10**	2.26	2.13	0.040	0.015	0.035	**0.000**	0.157	0.091
13	1230.31(4)	144/131	S1H4N5	**2.70**	5.95	4.94	**2.20**	3.77	3.04	**0.000**	0.040	0.052	**0.001**	0.081	0.087
14	1017.09(5)	144/131	H5N5F2	**4.50**	**5.44**	**5.14**	**3.70**	4.40	3.97	**0.003**	**0.003**	**0.002**	**0.000**	0.041	0.016
15	1045.75(4)	144/131	H5N2	**4.10**	4.20	**4.18**	1.60	2.43	2.01	**0.001**	0.005	**0.002**	0.133	0.188	0.220
16	1117.02(4)	144/131	H3N5	**2.90**	4.50	3.98	2.10	2.90	2.56	**0.001**	0.010	0.010	0.016	0.042	0.036
17	1157.54(4)	144/131	H4N5	**2.70**	4.16	3.71	**1.90**	2.92	2.47	**0.000**	0.025	0.022	**0.006**	0.074	0.071
18	1169.29(4)	144/131	S1H5N3	**3.60**	10.32	8.24	2.70	4.76	3.57	**0.004**	0.123	0.152	0.117	0.122	0.166
19	1216.04(4)	144/131	S1H4N4F1	**12.90**	34.60	27.90	6.70	11.30	9.27	**0.001**	0.221	0.238	0.018	0.254	0.221
20	1270.82(4)	144/131	S1H5N5	**2.70**	4.40	3.86	**2.00**	3.40	2.71	**0.000**	0.059	0.055	**0.003**	0.081	0.099
21	1220.05(4)	144/131	S1H5N4	1.30	2.71	2.28	**1.80**	3.00	2.45	0.267	0.025	0.052	**0.006**	0.067	0.080
21	976.23(5)	144/131	S1H5N4	1.70	**3.70**	3.08	**2.70**	3.32	**2.98**	0.067	**0.003**	0.008	**0.000**	0.033	0.016

Abbreviation: ACN, acetonitrile.

**FIGURE 2:**
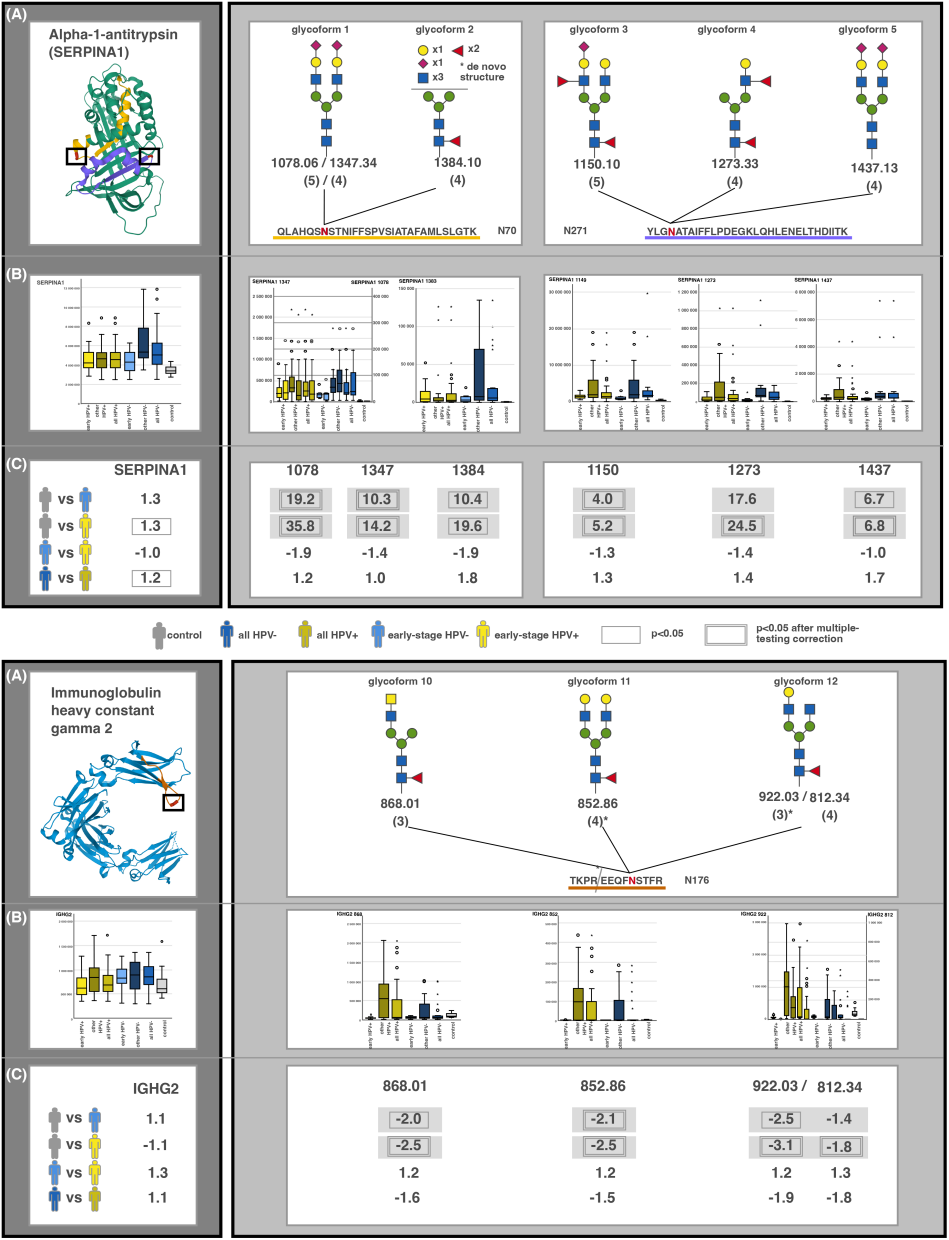
Examples of significantly different glycopeptides identified in alpha‐1‐antitrypsin (SERPINA1) (top) and immunoglobulin heavy constant gamma 2 (IGHG2) (bottom). The dark gray box on the left indicates the protein‐level and the light gray box on the right indicates the glycopeptide‐level data. Panel (A) on the left, the SERPINA1 protein and the corresponding 2 N‐glycosylation site on which significantly different glycopeptides are located. To the right, proposed structures of the significantly different glycopeptides, unregulated in early‐stage cancer. The peptide chain is shown with the N‐site shown in red. Panel (B) box plots of the SERPINA1 protein and its glycopeptides that are significantly different between OPSCC and controls. Each glycoform is separately labeled according to Table 2. Glycoform 2 (m/z 1384.1) had multiple feasible structures but did not match any known human glycan structures. Panel (C) The protein‐ and glycopeptide‐level changes and their significance. Each comparison is identified with regards to which group is compared to which. The FC for each comparison is shown. The surrounding boxes indicate whether the change reached statistical significance. SERPINA1‐glycopeptides are great examples of those that differentiate well between patients OPSCC and healthy controls. IGHG2 (below) represents one that despite passing statistical tests, this seems to be less accurate as the levels in healthy patients overlap completely with patients with OPSCC. When the peptide chain is shared between two proteins, as with haptoglobin (HP) and HP‐related protein, or Immunoglobulin heavy constant alpha 1/2, the glycopeptide chain could have been derived from either protein. In both SERPINA1 and IGHG2, we found the same glycopeptide ion with significantly different charge states 1078.06 (5) and 1347.34 (4) in SERPINA1 and 812.34 (4) and 922.03 (3) in IGHG2, which also had a missed cleavage. Levels of these different ion charge states were proportional to each other among the groups, as seen in the box plots here.

Alpha‐1‐antitrypsin (SERPINA1), that is, SERPINA1 is a glycoprotein of particular interest in our comparison. We identified 5 glycoforms from 2 different N‐glycosylation sites (N70 and N271) on SERPINA1. All of these glycoforms increased in early‐stage OPSCCs compared with controls (Figure 2), many of them significantly. Interestingly, these all were either core‐ and branch‐fucosylated or sialylated or both. Being up to 35 times more abundant in early‐stage OPSCCs, glycoform 1 distinguished between controls and low‐stage small OPSCC tumors, independent of HPV status (Tables S2 and S3).

Interestingly, the total protein expression of SERPINA1 was not altered in OPSCC versus controls; non‐significantly raised in HPV− (FC 1.3) and slightly elevated in HPV+ (FC 1.3), not significant when multiple‐testing correction was taken into account. Figure 2 further shows the variance of the SERPINA1 glycoform results in these samples, graphically demonstrating a narrow abundance range in the control group, but showing significantly larger abundance in the tumor groups. Figure 2 also shows suggested structures for the glycans, based on known human glycan structures.

It is interesting that in the early‐stage tumors there are fewer outliers and narrower confidence intervals, potentially reflecting the more homogenous states of the tumors while they are at an early‐stage. This may explain why the FCs and statistical significance of some glycoforms are not always as significant in the later‐stage tumor comparisons. Development of a tumor typically occurs through specific pathways, such as Pi3k‐akt–mtor signaling in HPV+ OPSCCs,^33^ and this may be a reflection of typical responses to known pathway aberrations in early‐stage tumors. As the tumor progresses, becomes less and less differentiated, with new mutations appearing as the tumor progresses, a reactive change in glycosylation in either the tumor environment or immune cells could contribute to glycosylation changes. Alternatively, as tumors progress this may affect lifestyle factors such as diet, given the location of the tumor in the oropharynx in this series, which may in turn affect glycosylation patterns seen in the plasma.

SERPINA1 is known to have a role as an acute‐phase reactant and immunomodulator, is produced by hepatocytes but also immune cells and affects and is regulated by various chemokines.^34^ Variations in its glycosylation patterns have been demonstrated in various cancers, among others lung, liver, and breast carcinomas,^35^, ^36^, ^37^ notable glycan alterations included increased levels of the sLex epitope and increased levels of core fucosylation.^38^ Three of our five SERPINA1 glycoforms that were upregulated in the patients with OPSCC were core‐fucosylated, as determined by manual curation of the spectra, identifying fragments of an intact peptide with N‐acetylhexosamine (HexNAc) as well as fucose. Within our methodological restrictions, we were able to determine relative abundance, composition, and likely structure of the glycoforms. Compared to other studies that also assessed intact N‐glycopeptides, our glycoforms 9, 21 were non‐significantly downregulated in pancreatic cancer, glycoforms 6 and 13 were non‐significantly upregulated in pancreatic cancer, and glycoform 21 non‐significantly downregulated in oral squamous cell carcinoma.^23^, ^24^ In these studies, as well as our current study however, the glycopeptides targeted for identification were those that were statistically significant between groups; therefore, we cannot see the whole glycosylation profile.

Haptoglobin (HP) is another acute‐phase protein, crucial for the neutralization of oxidative damage as well as elimination of free hemoglobin. In our study, there was a slight increase in the abundance of certain glycoforms occupying two N‐glycosylation sites. Interestingly, in the case of glycoforms with occupancy at site 241, that were significantly overexpressed in cancer, their base proteins were also overexpressed a similar amount. This suggests that in this case the overexpression of the glycoforms is due to the increase in base‐protein levels. This protein overexpression and with it, glycoform overexpression could represent a glycoform performing a basic function for example involved in protein folding or functional capacity to neutralize oxidative damage.

With N‐glycopeptide site 184, glycoform 9 of HP was of interest, as it was significantly increased in the early‐stage HPV+ tumors and higher‐stage HPV− tumors—to a greater extend that the increase in HP protein overexpression. This is a double sialylated glycan with the same composition as the glycan on glycoform 1. Increases in fucosylation and sialylation of HP have been noted in nonsmall cell lung cancer^39^ and increased fucosylation in pancreatic cancer and ovarian cancer^40^, ^41^; however unlike our study, there was no information about the N‐glycosylation site or of the proposed glycopeptide structures involved in these studies.

Immunoglobulin heavy constant gamma 2 (IGHA1/2) showed a significant decrease in 3 glycoforms in early‐stage HPV+ tumors, and in glycoform 11 in early‐stage HPV− tumors compared with controls, while the base protein remained stable. In Figure 2, box and whisker plots demonstrate the variability shown here again, with early‐stage tumors having very low homogenous values, and later‐stage tumors varying dramatically in their results.

We further analyzed HPV+ OPSCC compared to HPV− OPSCC, assessing whether there are clear differences attributed to the differing etiology. PCA analysis demonstrated significant overlap when comparing HPV+ and HPV− tumors with each other (Figure S1). Although there were significant glycovariant differences found between HPV+ and HPV− tumors, particularly in the IGHA1/2 protein, the differences were similar to the protein‐level expression changes. This suggests similarities of the states, and perhaps the physiological response to a small tumor derived from the same cell type in the same anatomical location.

On IGHA1/2, the upregulated glycoforms in both HPV+ and HPV− OPSCCs proposed structures (Figure S4) are mostly bisecting or sialylated or both. Their FCs range from 1.9 to 12.9 in HPV− and from 1.9 to 6.7 in HPV+ early‐stage OPSCCs. The protein FC increase of 1.5× in early‐stage HPV− and stable protein levels in early‐stage HPV+ tumors suggests, again, that the increased N‐glycosylation is independent of protein levels. Glycoform 19, which has a FC of 12.9, is a likely core‐fucosylated glycoform.

In this study, we perform proteomics using the serum processing as with the glycopeptidomics, differing from the norm of removing the top 12 or top 14 serum proteins, in order to directly compare the abundances of the protein‐level changes and glycopeptide‐level changes. Typically, in proteomic research the most abundant serum proteins are preliminarily depleted from the sample to improve the dynamic range of the study, allowing for identification and quantification of hundreds or even thousands of lower‐abundance serum proteins. However, the top 12 most abundant serum proteins are glycosylated. For the purpose of identifying important serum glycopeptides, in order to maximize clinical utility of this technology, we did not deplete the most abundant serum proteins from the samples, removing only albumin. As we did not remove the top 12/14 most abundant proteins, as expected we identified fewer proteins than is typical of MS research due to decreased dynamic range. We identified 76 proteins in total, of which 52 are glycoproteins. These are shown in Table S4.

Various enrichment strategies have been utilized due to various challenges in identifying and quantifying glycopeptides due to both low abundance and low proton affinity, thus challenges in identification in the MS methodology.^42^ Here, we opted to enrich our samples using size‐exclusion chromatography, as it enhances the detection of N‐glycopeptides without selecting for specific glycan features such as fucosylation or sialylation.^43^ It takes advantage of glycopeptides being larger than peptides, as opposed to being influenced by the protein backbone or type of glycan.^42^

As these are serum proteins, it is unclear from where they were secreted, whether from the tumor itself, cells surrounding the tumor, the liver, the leukocytes, or other cells.

Gender distribution is not equal in the control and case groups; however, we performed PCA analysis comparing male versus female samples, finding no separation between the groups (Figure S1). Lifestyle changes are one thing to affect glycosylation, but due to the nature of the study we were unable to acquire lifestyle data from the controls and thus could not use it as a variable.

Given the complexity of glycoproteins, different approaches can be used to identify different aspects of their form or structure. Our approach is limited in that, where glycan alterations are found at different glycosylation sites within the same protein, we are unable to determine whether these occur together within a protein, or separately. Additionally, we cannot determine types of glycosidic bonds. Although knowledge of individual glycans attached to peptides provides more information about the state of the corresponding protein expression and about the state and pattern of glycosylation, analysis of total glycomics would still be beneficial, and can complement the glycopeptidomics analysis that we have performed here. Analysis of total glycomics may additionally offer insights about both the activity of glycosyltransferases and the availability or lack of specific sugars and/or glycans. This would be an important avenue to pursue in future research on the topic. Further limitations of this work include the inability to differentiate further post‐translational modifications such as sulphated glycans; however, the raw glycopeptide composition with a proposed structure alone gives a good idea of the surface of glycoprotein is altered. Furthermore, the functions of individual glycan/protein combinations are not well understood.

Overall, in early‐stage OPSCCs, the significant glycovariance that we identified seems to be independent of the protein levels. Compared with controls, the majority of the significantly differently expressed glycoforms found in HPV+ and HPV− OPSCCs are similar. Interestingly, although the majority of glycoforms that were altered in this series of early‐stage OPSCCs were likely complex and biantennary, other features such as fucosylation, sialylation, and bisection varied by base protein. The glycotransferases do not possess base‐protein specificity,^4^ so this may be an indication of another, local cell type‐level alteration.

We are the first to characterize and identify differentially expressed glycoforms in OPSCC compared to healthy individuals, where there is no change in the protein expression between groups. The alterations in the SERPINA1 glycovariants that we identified would be worth assessing these changes in other head and neck cancers as well as other cancers in general, to determine the utility of such findings for diagnostic purposes. Many of the currently used diagnostic biomarkers in other cancers (e.g., alpha‐fetoprotein) have elevated levels in other cancers as well; however, the differences in the glycovariants may add to the specificity of the tests, when compared to template alterations such as proteins. We elucidated the compositions of these OPSCC glycoforms, that otherwise have not yet been identified, that will pave the way for future research on the topic.

## 4 CONCLUSIONS

We have elucidated the composition glycoforms that are significantly abnormal in OPSCC, up to 35x more abundant, even in early‐stage tumors. Further research is needed to determine whether these glycoforms could be used for example in cancer screening. This study gives insight into increases in N‐glycosylation levels despite protein expression remaining stable. Such highly sensitive glycopeptide changes indicate that glycovariance may be more beneficial than proteomic analysis, which thus far has not been fruitful in producing diagnostic cancer biomarkers. Furthermore, adding to the knowledge of glycosylation changes in OPSCC brings us closer to someday applying glycosylation targeted inhibitors in treatment of these diseases.

## AUTHOR CONTRIBUTIONS

Amy Dickinson, Sakari Joenväärä, Antti Mäkitie, and Suvi Silén; contributed to the study concept, design and planning phase. Petri Mattila; collected the patient samples. Timo Carpén; collected the clinical information. Amy Dickinson and Tiialotta Tohmola; performed sample preparation supervised by Sakari Joenväärä; The mass spectrometry process was performed and overseen by Sakari Joenväärä; Mass spectrometry analysis was performed by Amy Dickinson, Jutta Renkonen, Tiialotta Tohmola, and Sakari Joenväärä; Statistical analysis was performed by Sakari Joenväärä and Amy Dickinson; MaxEnting was performed by Tiialotta Tohmola, Amy Dickinson, and Jutta Renkonen; Glycopeptide identification analysis was performed by Sakari Joenväärä; Results interpretation was performed by Amy Dickinson, Sakari Joenväärä, Suvi Silén, and Antti Mäkitie; Manuscript preparation was performed by Amy Dickinson, Sakari Joenväärä, and Suvi Silén. All authors played a role in revision of the manuscript and have approved the final version.

## CONFLICT OF INTEREST STATEMENT

None to declare.

## ETHICS STATEMENT

The study plan was approved by the institutional Research Ethics Board at the Helsinki University Hospital (DNr. 51/13/03/02/2013). The study was performed in accordance with the Declaration of Helsinki.

## CONSENT TO PARTICIPATE

Written informed consent was obtained from all patients.

## Supporting information


Data S1:

Figure S1.

Figure S2.

Figure S3.

Figure S4.

Table S1.

Table S2.

Table S3.

Table S4.

## Data Availability

Data are available via ProteomeXchange with identifiers PXD016709 and PXD016727.
