# Supplementary figures and images for: Altered microheterogeneity at several N‐glycosylation sites in OPSCC in constant protein expression conditions
Source: FASEB Bioadv. 2023 Dec 14;6(1):26–39. doi: 10.1096/fba.2023-00066 (PMC10782471; doi:10.1096/fba.2023-00066)

Principal Components Analysis

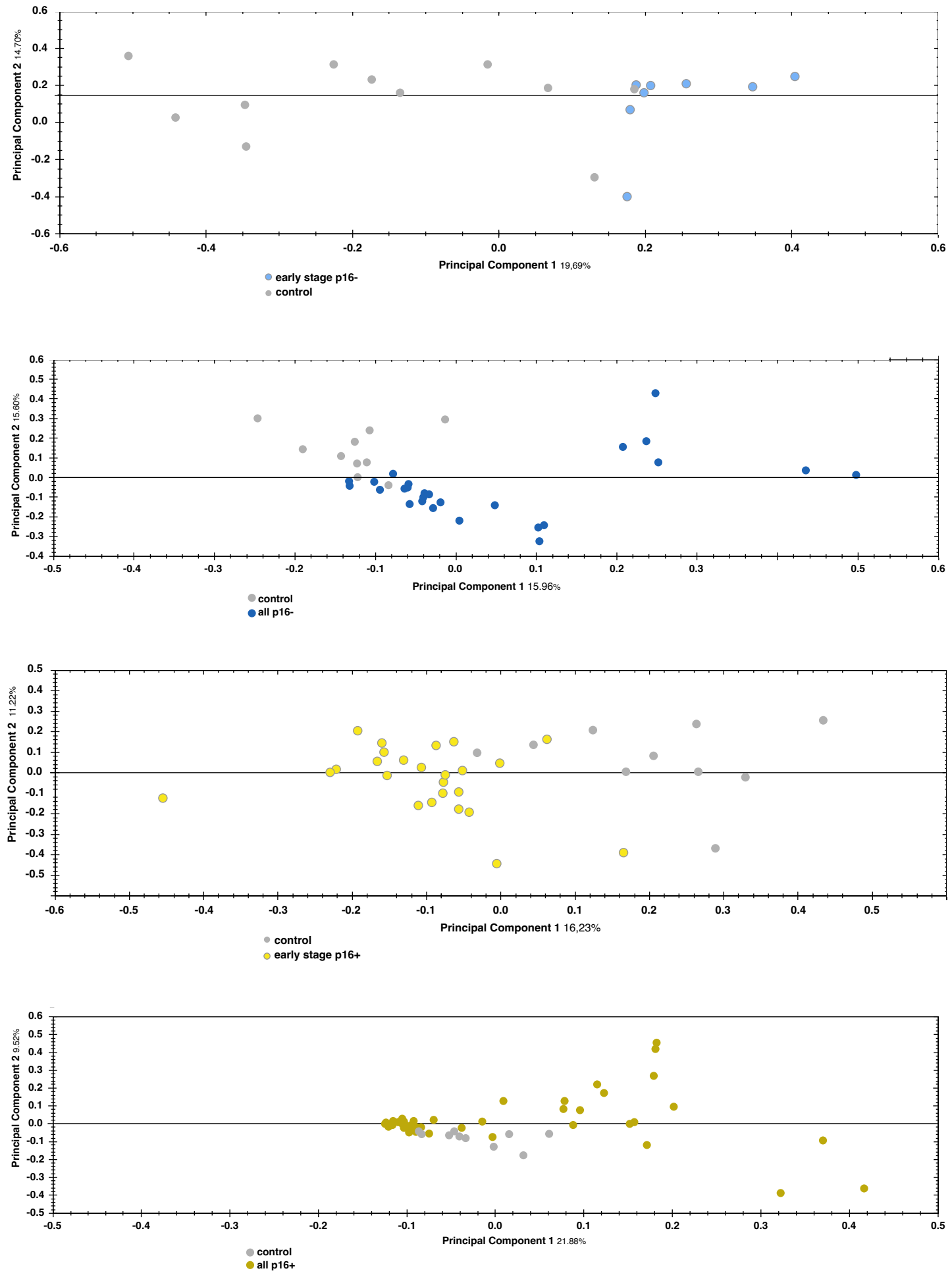

Principal Components Analysis

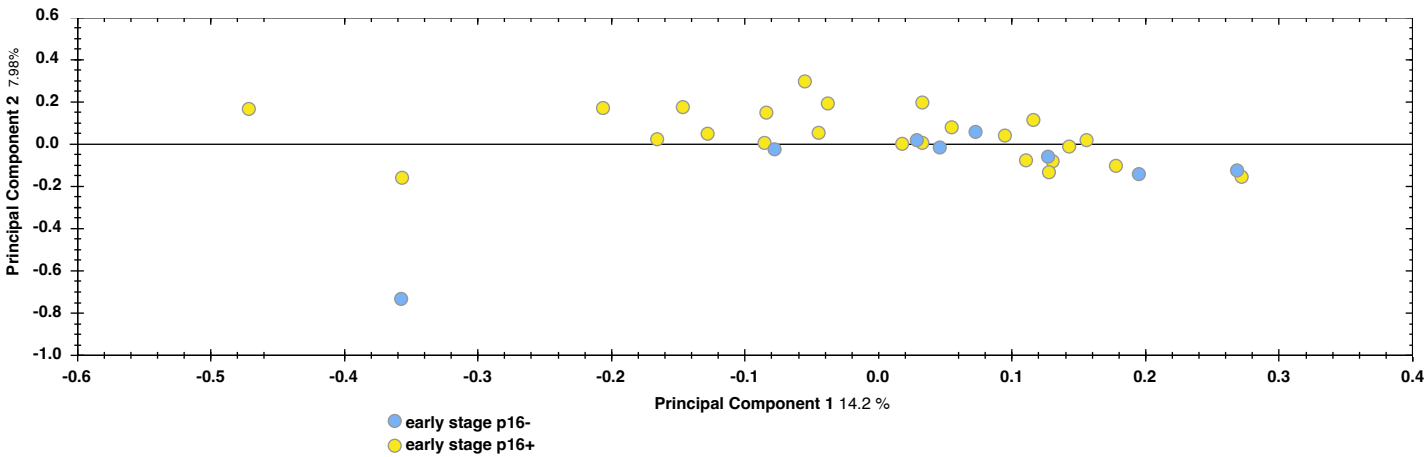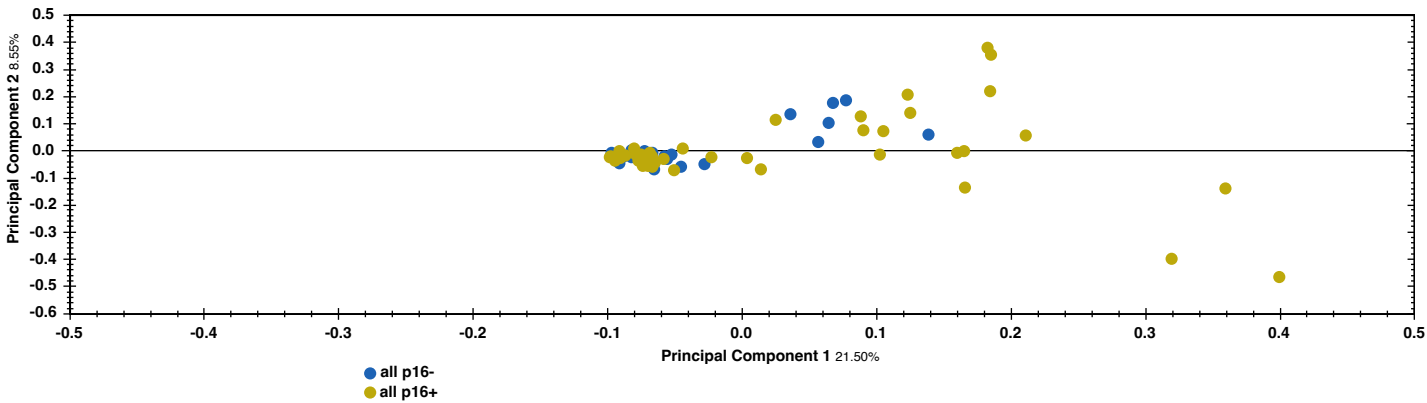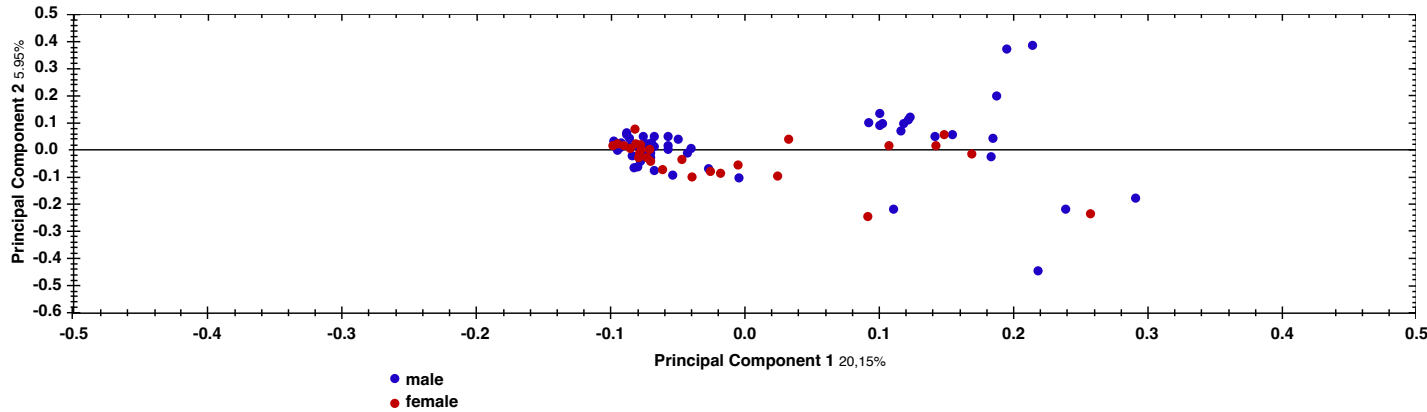

Supplement: Supplementary file 2 — Figure S1. [file FBA2-6-26-s007.pdf]

Principal Components Analysis

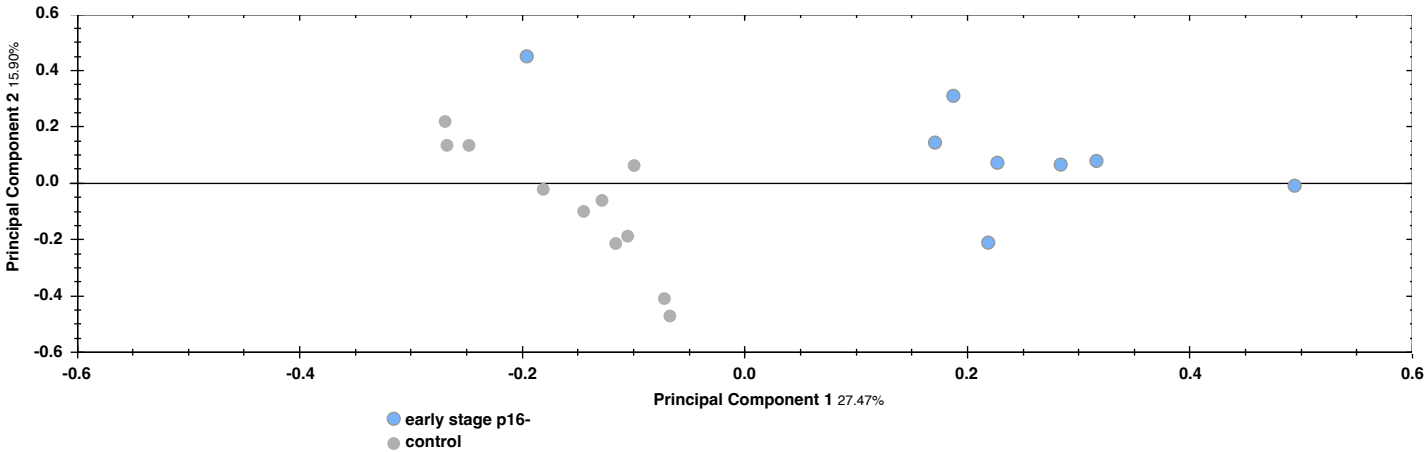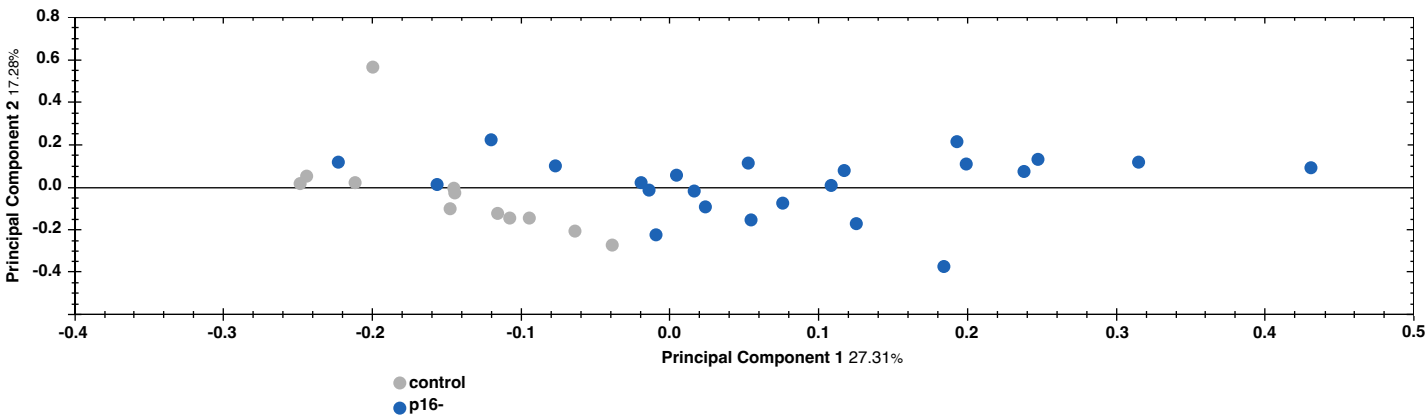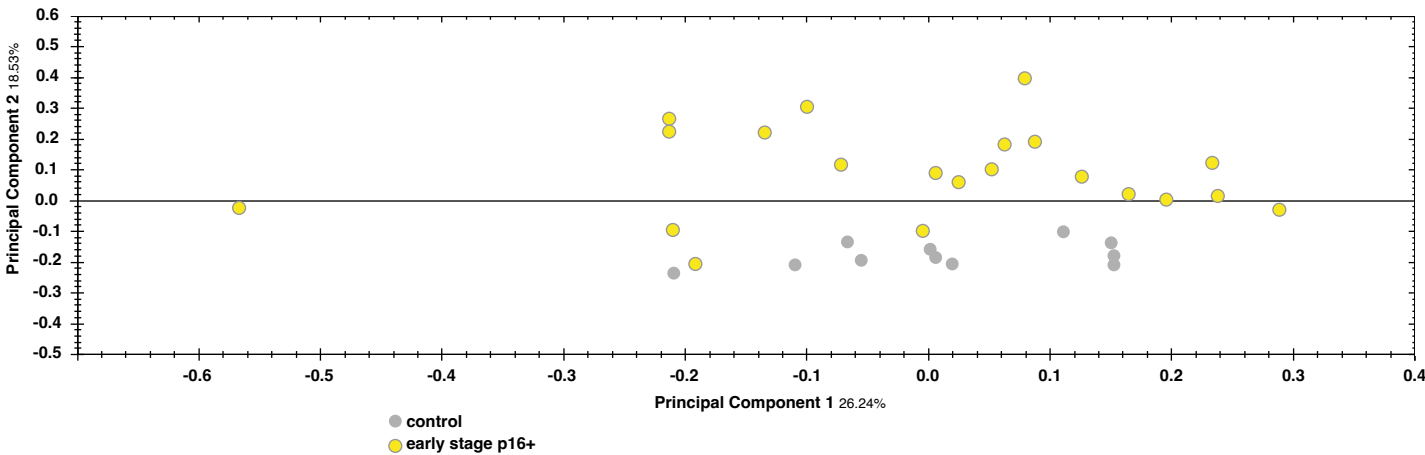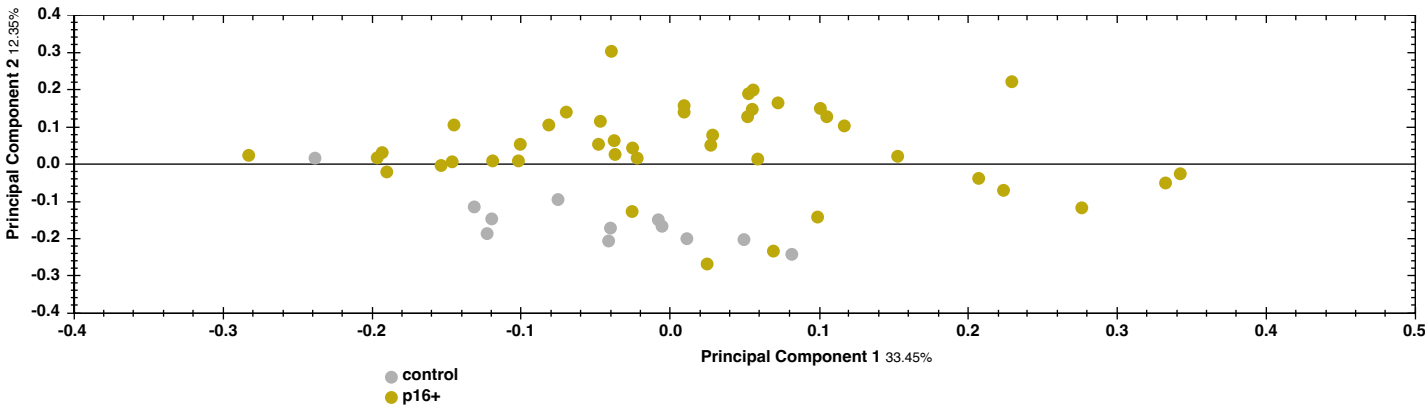

Principal Components Analysis

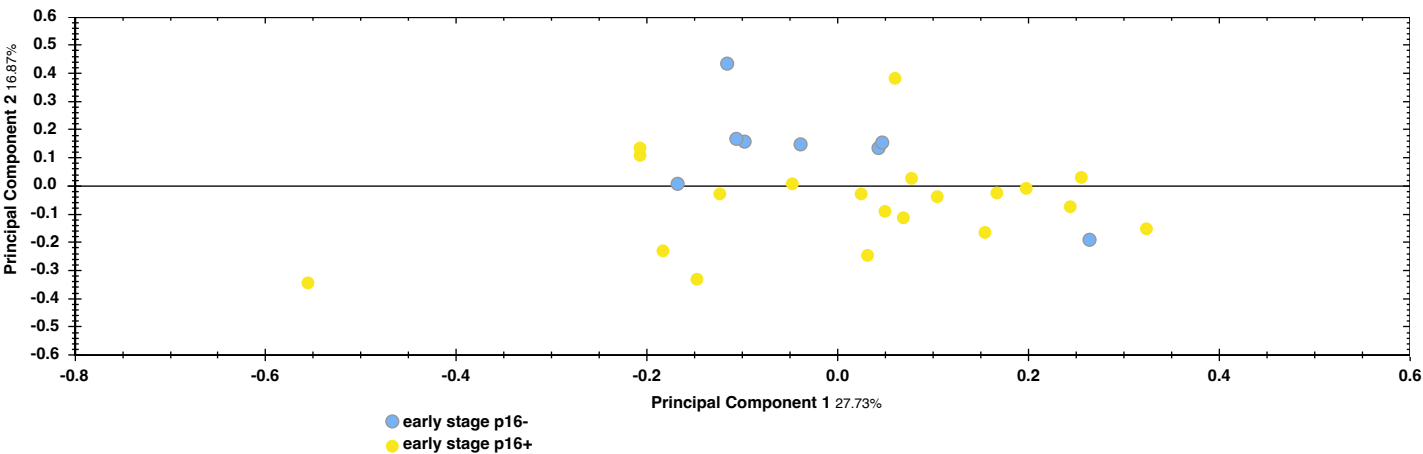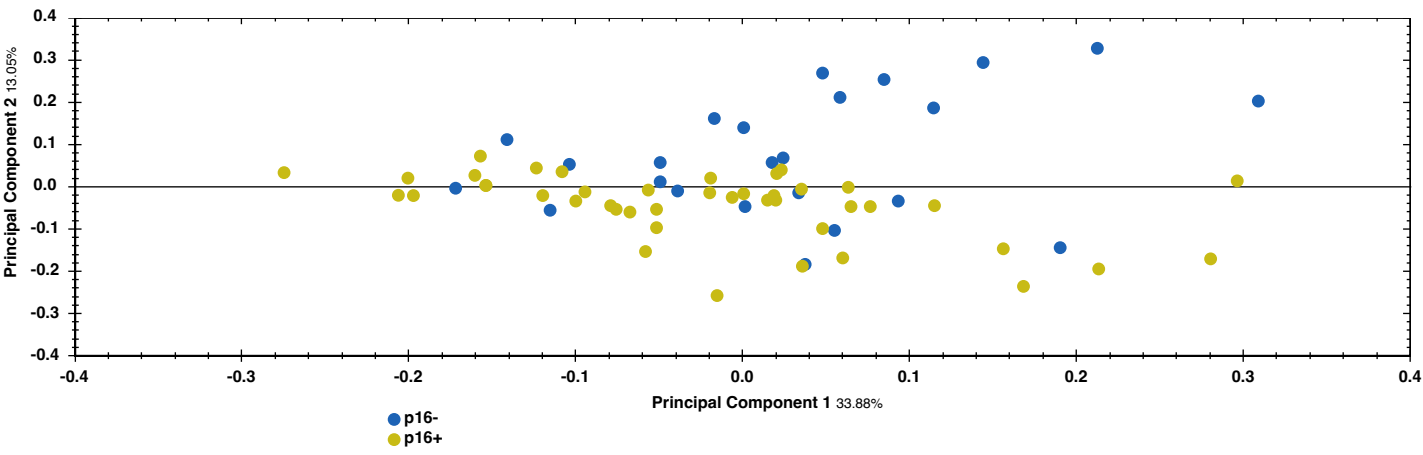

Supplement: Supplementary file 4 — Figure S3. [file FBA2-6-26-s004.pdf]

FIGURE S4

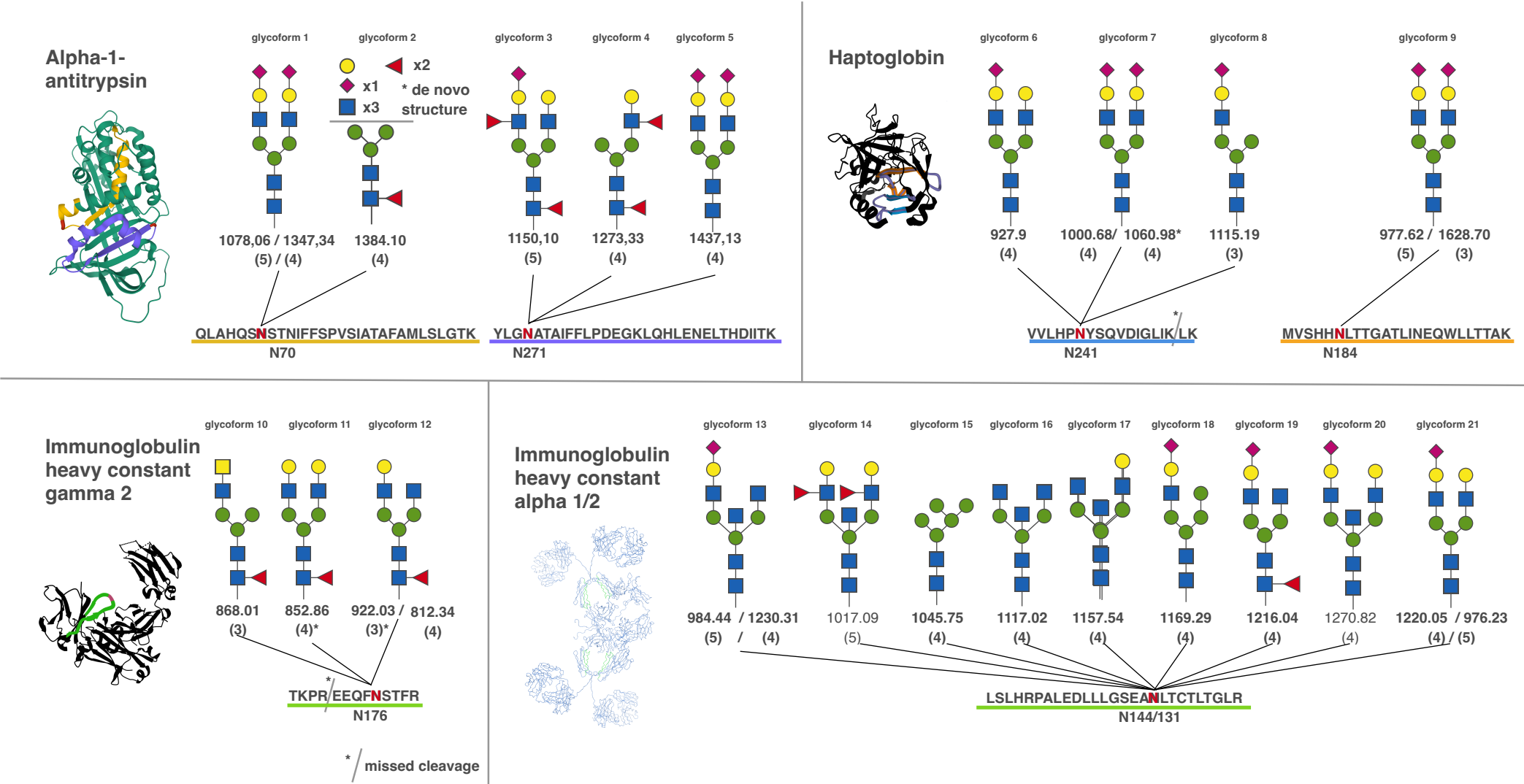

Supplement: Supplementary file 5 — Figure S4. [file FBA2-6-26-s008.pdf]
