# Supplementary material for: Altered microheterogeneity at several N‐glycosylation sites in OPSCC in constant protein expression conditions
Source: FASEB Bioadv. 2023 Dec 14;6(1):26–39. doi: 10.1096/fba.2023-00066 (PMC10782471; doi:10.1096/fba.2023-00066)
Supplement: Supplementary file 3 — Figure S2. [file FBA2-6-26-s003.pdf]

m/z 1150.1011 charge 5 scan 0–0

Score= 157.71 , Hits= 50 , Explained Intensity= 0.47

Peptide: A1AT_HUMAN[268,298]:YLGNATAIFFLPDEGKLQHLENELTHDIITK

Glycan: SHNH(FHNH)HN(F)N, S1H5N4F2

Charge: 5H

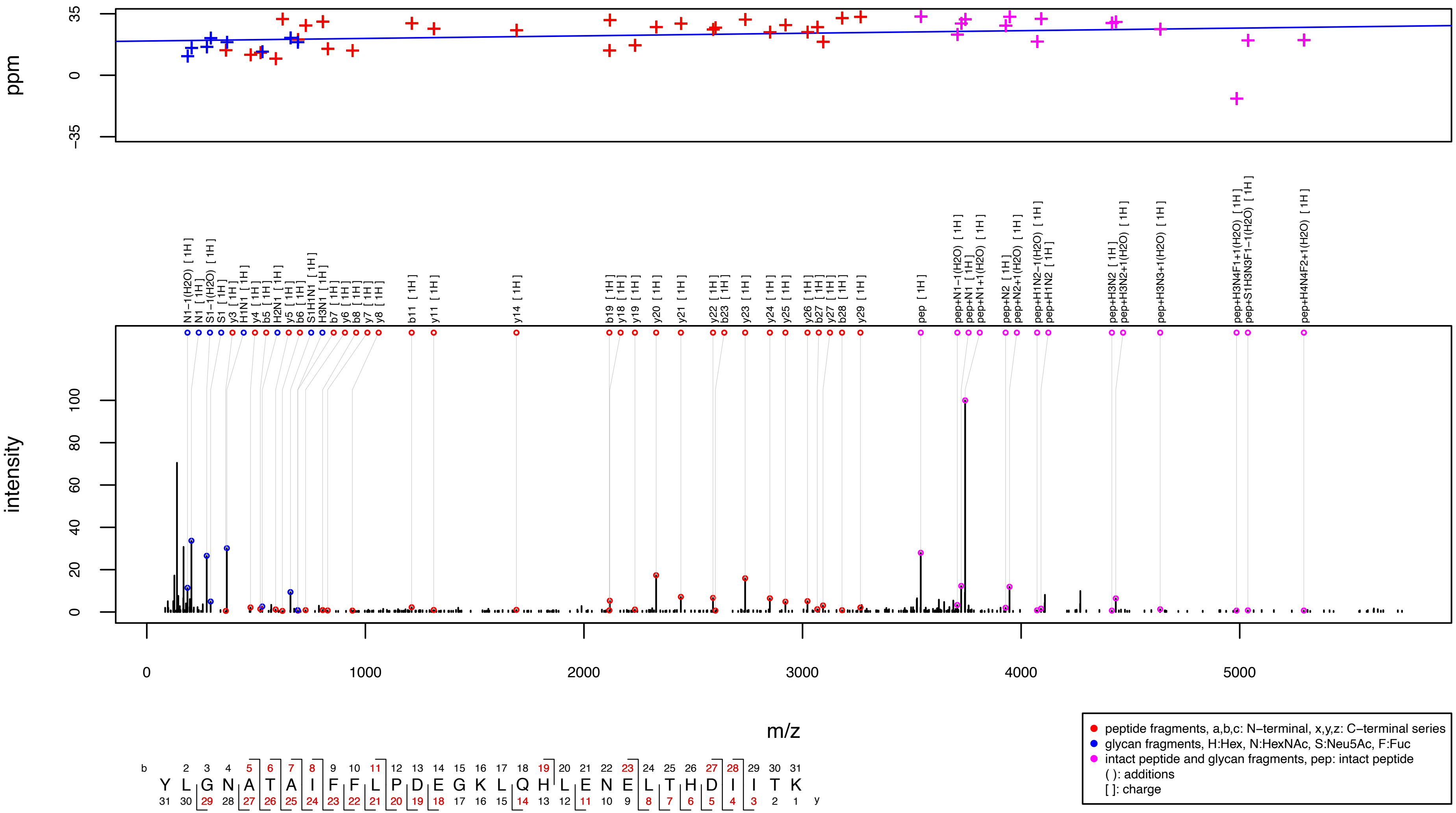

Glycopeptide ID – *Mon Dec 2 18:05:44 2019*

Supplementary Figure 2 GP annotations page 1/27

m/z 1169.2875 charge 4 scan 0–0

Score= 139.42 , Hits= 44 , Explained Intensity= 0.4

Peptide: IGHA1_HUMAN[127,153]:LSLHRPALEDLLLGSEANLTCTLTGLR

Glycan: SHNH(HH)HNN, S1H5N3

Charge: 4H

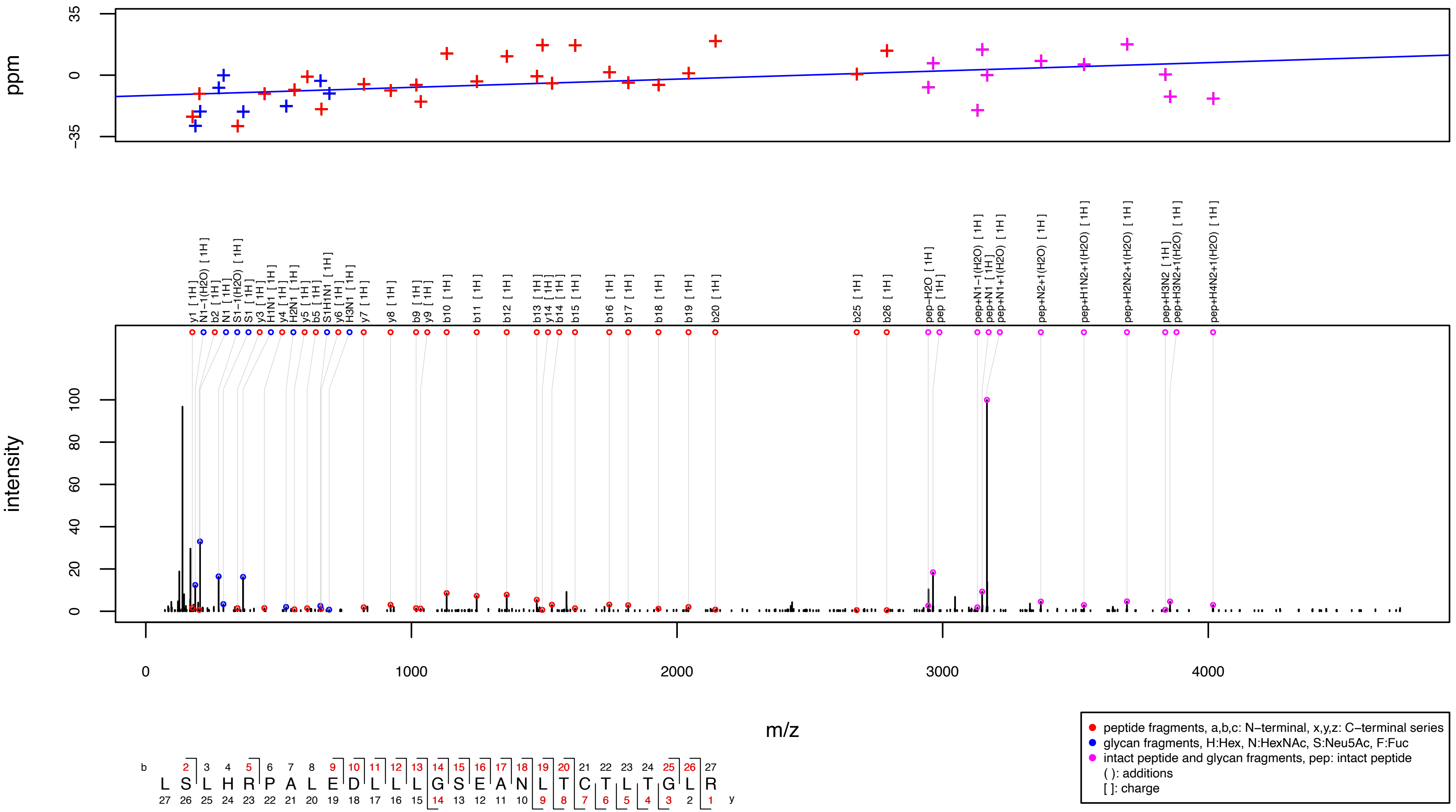

Glycopeptide ID – *Mon Dec 2 18:05:44 2019*

m/z 1157.538 charge 4 scan 0–0

Score= 132.89 , Hits= 41 , Explained Intensity= 0.35

Peptide: IGHA1_HUMAN[127,153]:LSLHRPALEDLLLGSEANLTCTLTGLR

Glycan: HNH(NH)(N)HNN, H4N5

Charge: 4H

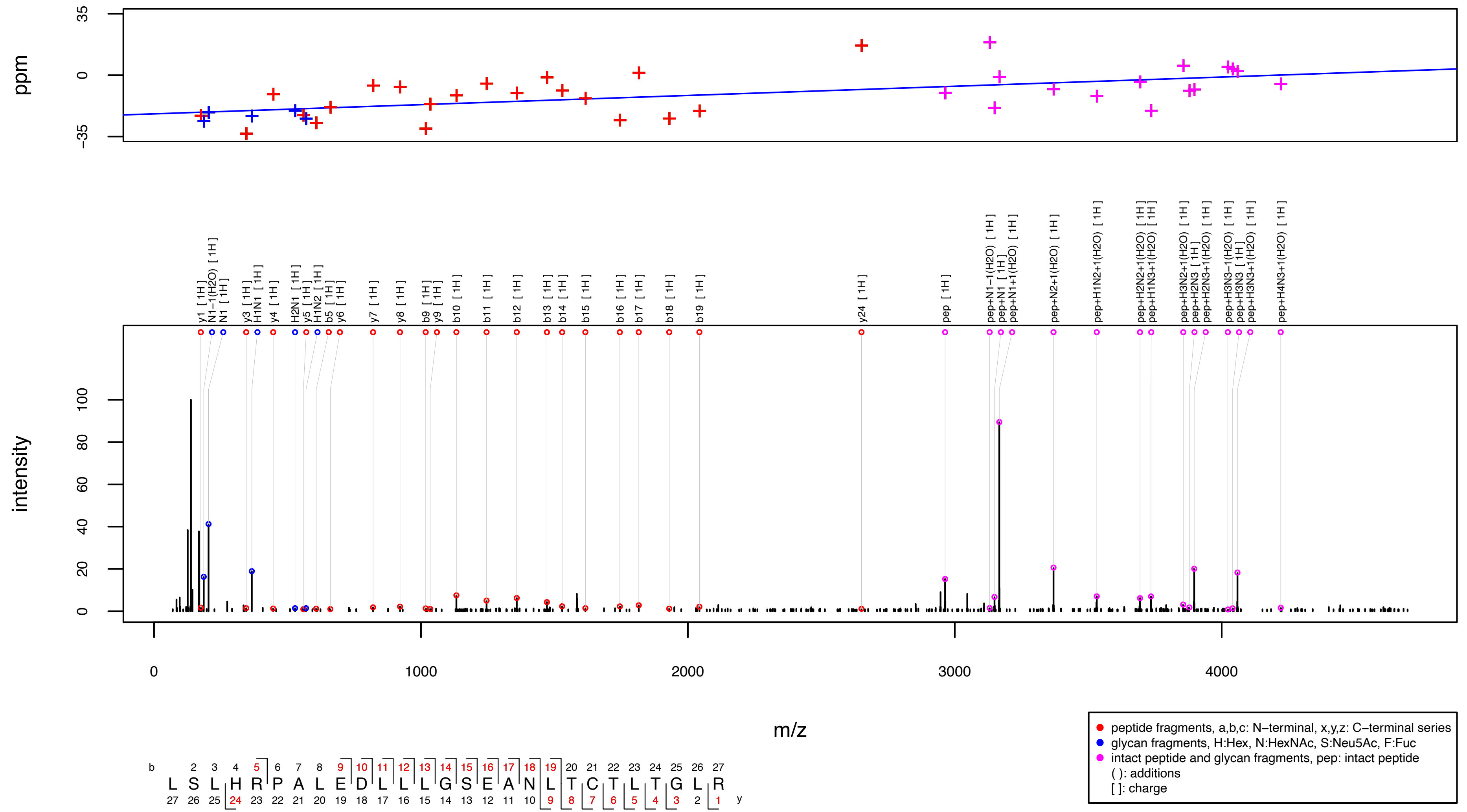

Glycopeptide ID – *Mon Dec 2 18:05:53 2019*

m/z 927.9 charge 4 scan 0–0

Score= 104.2 , Hits= 33 , Explained Intensity= 0.22

Peptide: HPT_HUMAN[236,251]:VVLHPNYSQVDIGLIK

Glycan: SHNH(HNH)HNN, S1H5N4

Charge: 4H

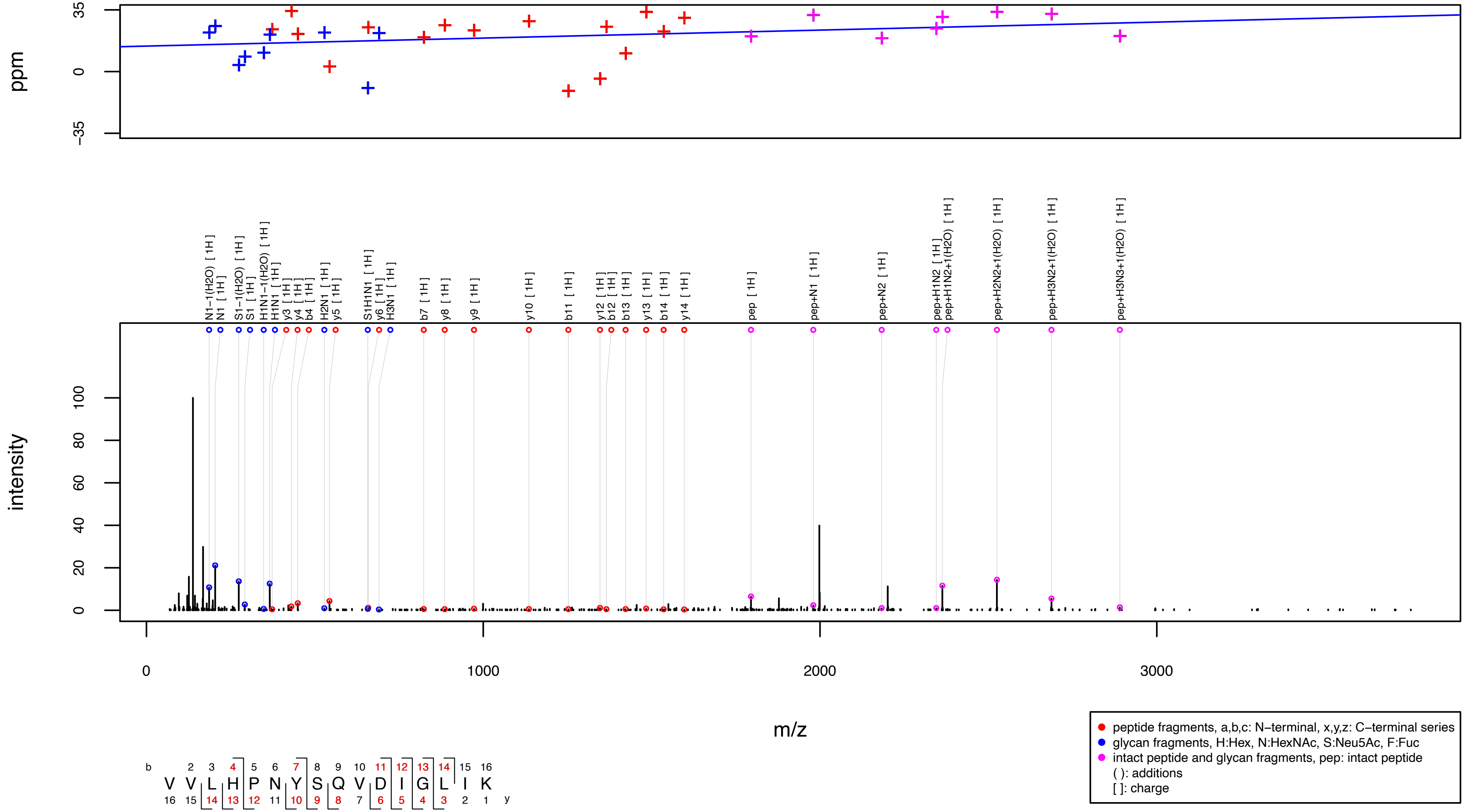

Glycopeptide ID – *Mon Dec 2 18:06:01 2019*

m/z 977.6183 charge 5 scan 0–0

Score= 177.07 , Hits= 53 , Explained Intensity= 0.31

Peptide: HPTR_HUMAN[121,144]:MVSHHNLTTGATLINEQWLLTTAK

Glycan: SHNH(SHNH)HNN, S2H5N4

Charge: 5H

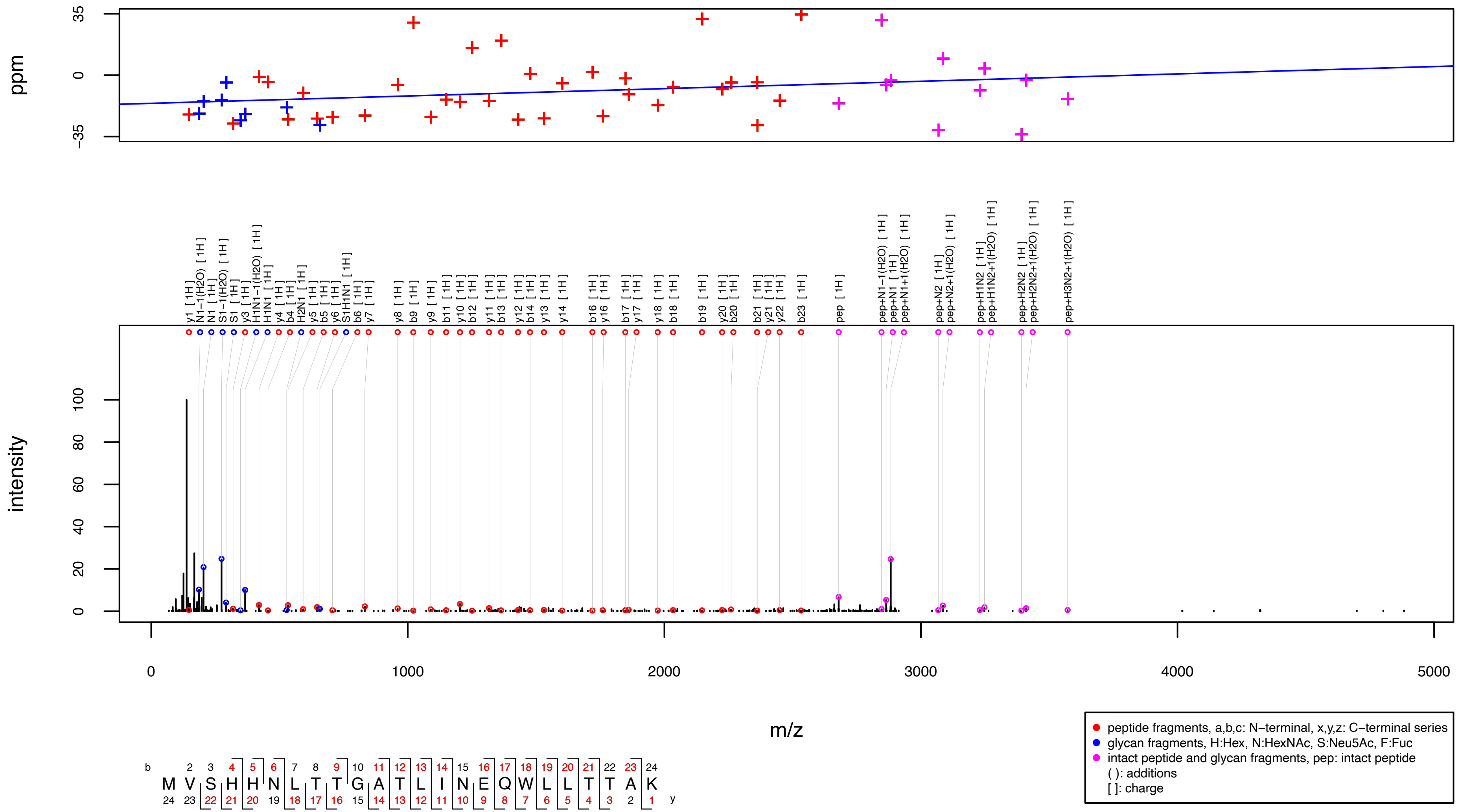

Glycopeptide ID – *Mon Dec 2 18:05:41 2019*

m/z 1045.7515 charge 4 scan 0–0

Score= 92.28 , Hits= 30 , Explained Intensity= 0.22

Peptide: IGHA1_HUMAN[127,153]:LSLHRPALEDLLLGSEANLTCTLTGLR

Glycan: H(H)H(H)HNN, H5N2

Charge: 4H

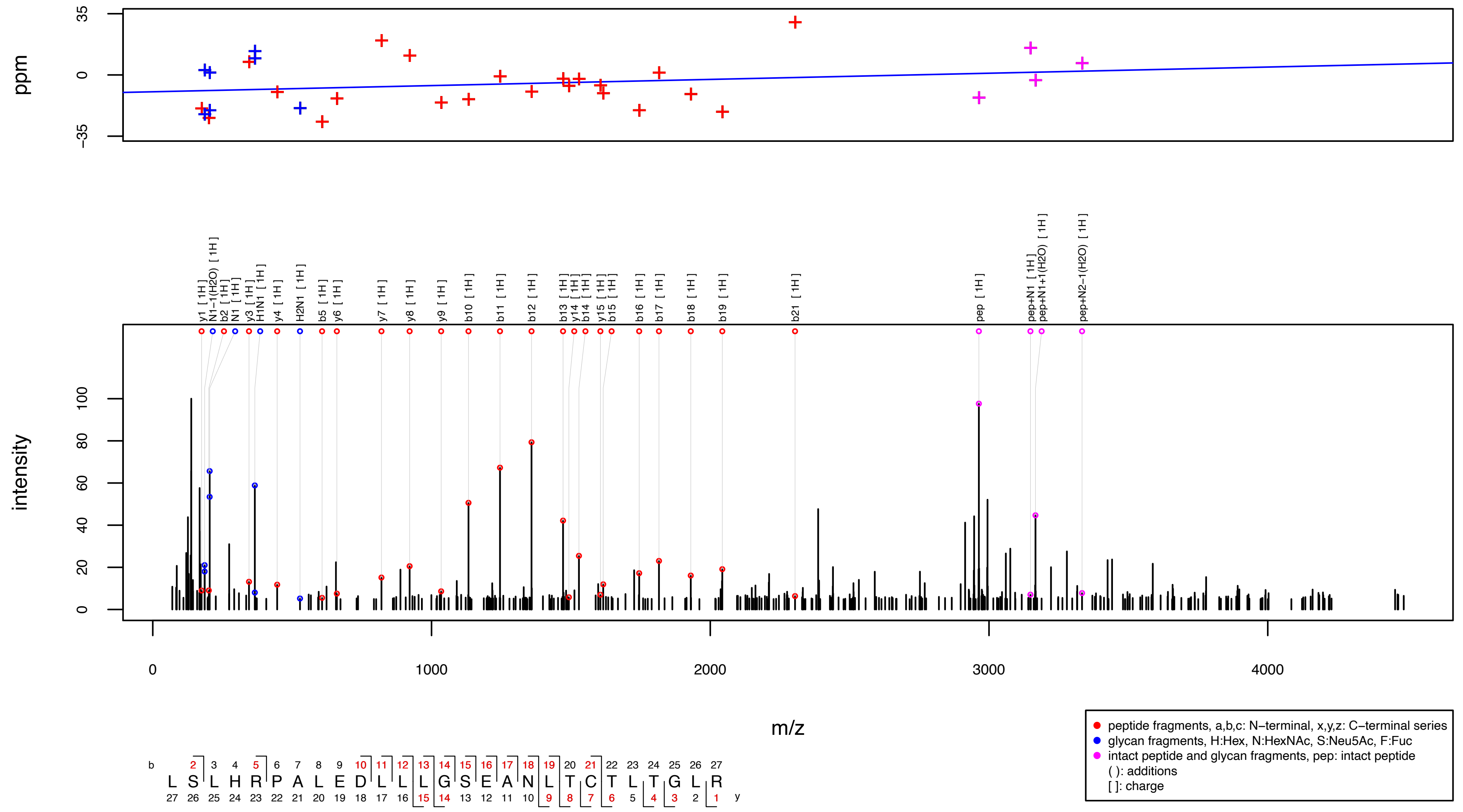

Glycopeptide ID – *Mon Dec 2 18:05:50 2019*

m/z 1628.6963 charge 3 scan 0–0

Score= 228.04 , Hits= 70 , Explained Intensity= 0.64

Peptide: HPTR_HUMAN[121,144]:MVSHHNLTTGATLINEQWLLTTAK

Glycan: SHNH(SHNH)HNN, S2H5N4

Charge: 3H

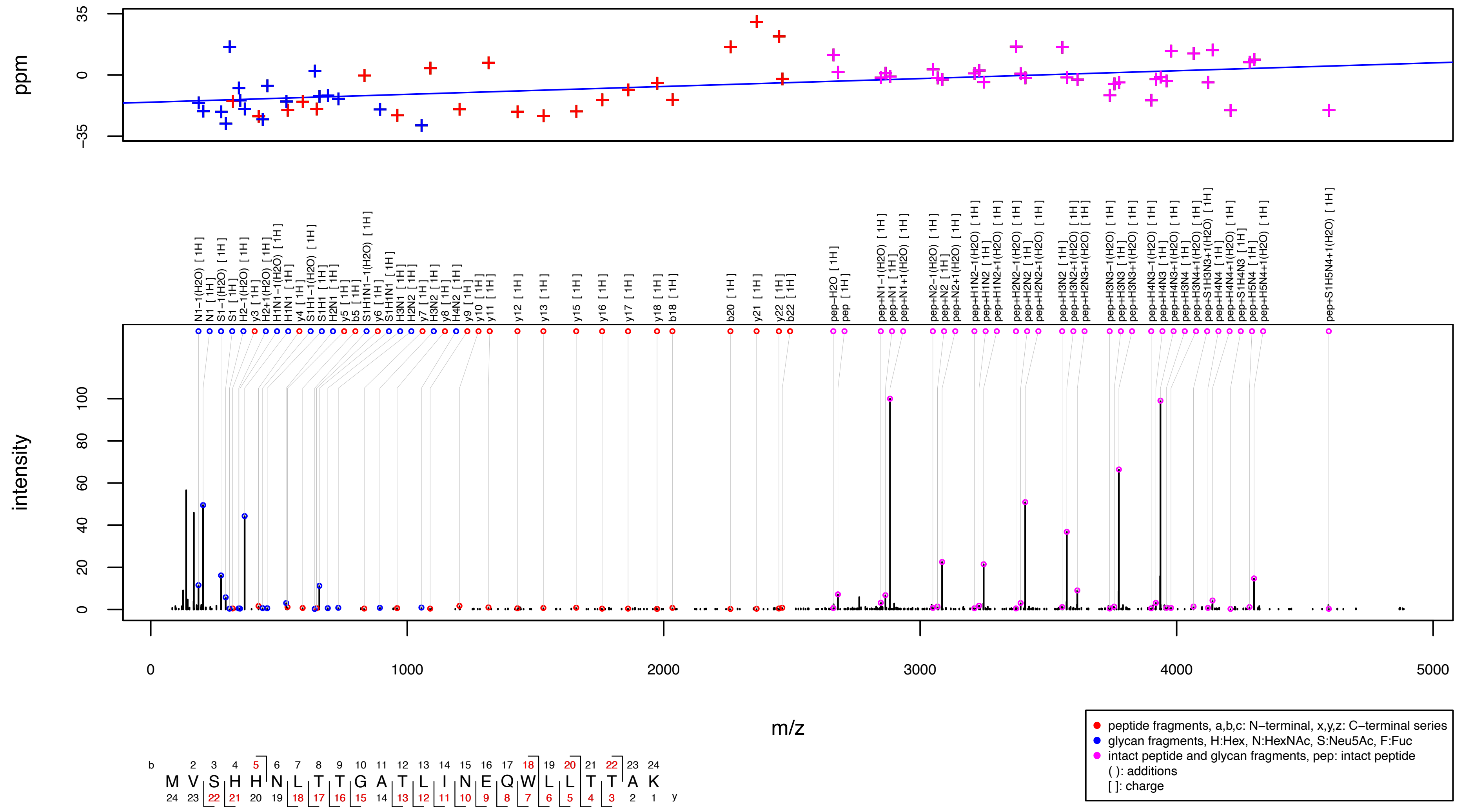

m/z 1437.1254 charge 4 scan 0–0

Score= 208.08 , Hits= 62 , Explained Intensity= 0.51

Peptide: A1AT_HUMAN[268,298]:YLGNATAIFFLPDEGKLQHLENELTHDIITK

Glycan: SHNH(SHNH)HNN, S2H5N4

Charge: 4H

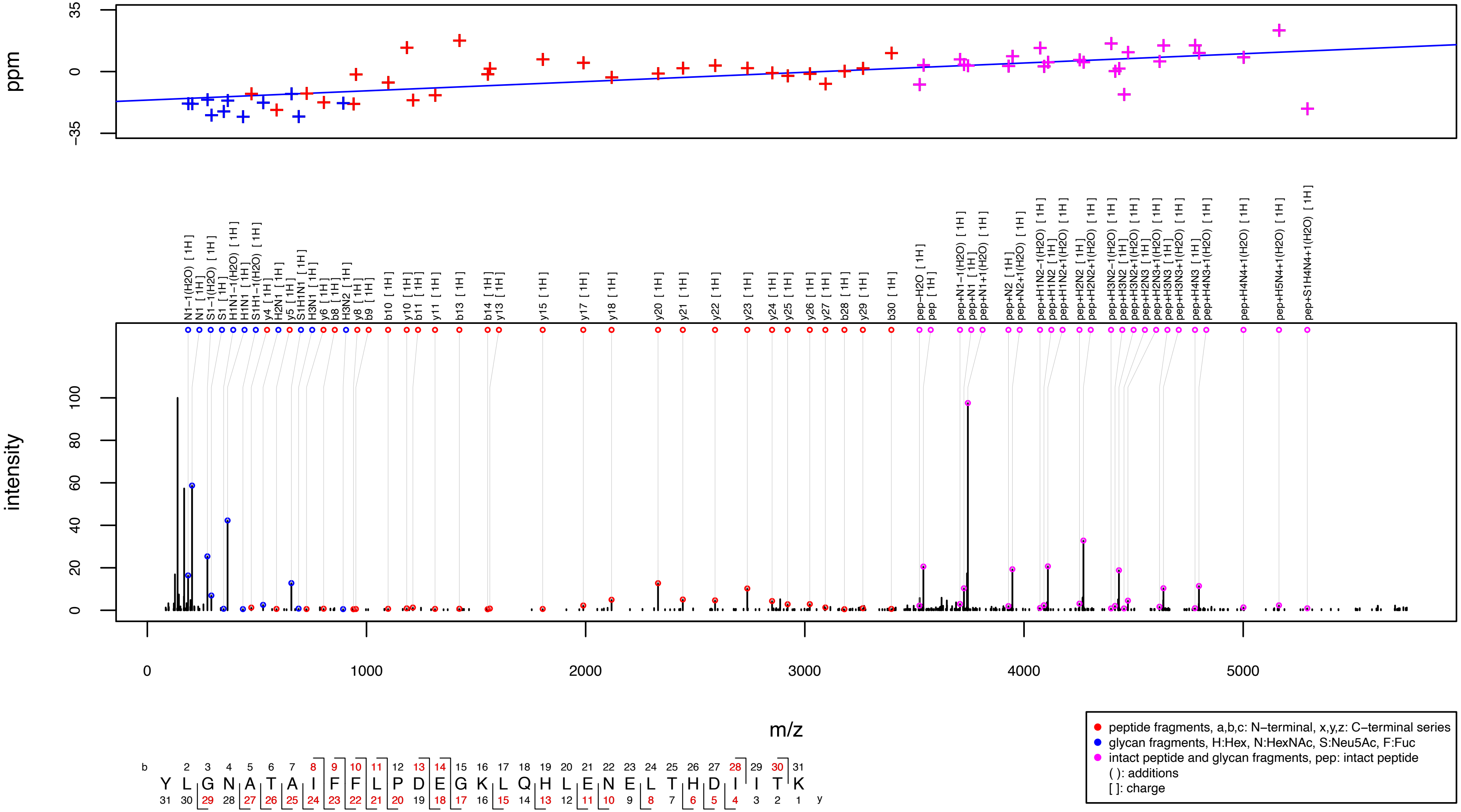

- peptide fragments, a,b,c: N−terminal, x,y,z: C−terminal series
- glycan fragments, H:Hex, N:HexNAc, S:Neu5Ac, F:Fuc
- intact peptide and glycan fragments, pep: intact peptide
- ( ): additions
- [ ]: charge

Glycopeptide ID – *Mon Dec 2 18:05:43 2019*

m/z 1115.1879 charge 3 scan 0–0

Score= 117.97 , Hits= 36 , Explained Intensity= 0.43

Peptide: HPT_HUMAN[236,251]:VVLHPNYSQVDIGLIK

Glycan: SHNH(H)HNN, S1H4N3

Charge: 3H

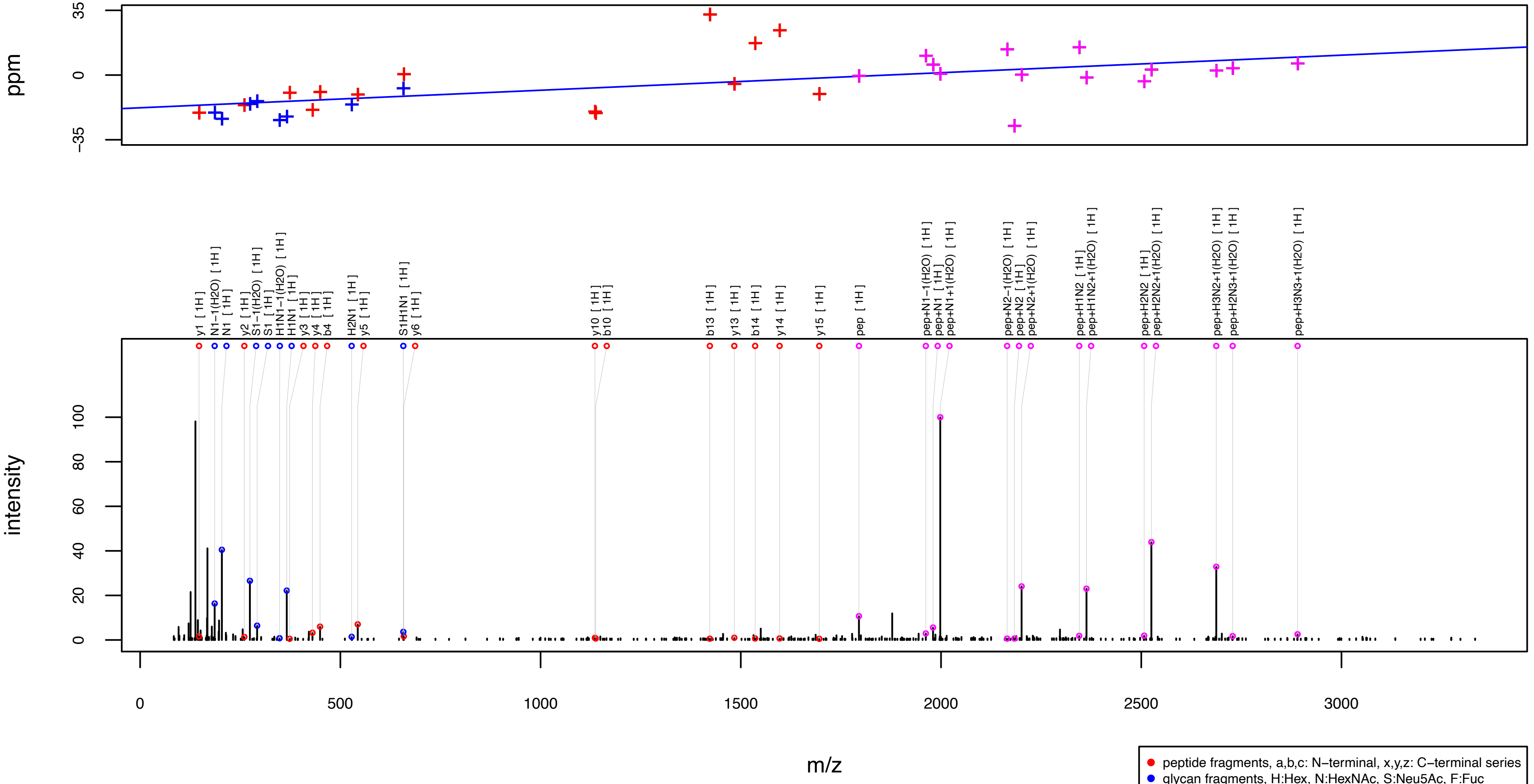

b 2 3 4 5 6 7 8 9 10 11 12 13 14 15 16

V V L H P N Y S Q V D I G L I K

16 15 14 13 12 11 10 9 8 7 6 5 4 3 2 1 y

- peptide fragments, a,b,c: N−terminal, x,y,z: C−terminal series
- glycan fragments, H:Hex, N:HexNAc, S:Neu5Ac, F:Fuc
- intact peptide and glycan fragments, pep: intact peptide
- ( ): additions
- [ ]: charge

Glycopeptide ID – *Mon Dec 2 18:06:04 2019*

m/z 1384.0969 charge 4 scan 0–0

Score= 97.91 , Hits= 40 , Explained Intensity= 0.23
Peptide: A1AT_HUMAN[64,93]:QLAHQSNSTNIFFSPVSIATAFAMLSLGTK
Glycan: S1H5N4F3
Charge: 4H

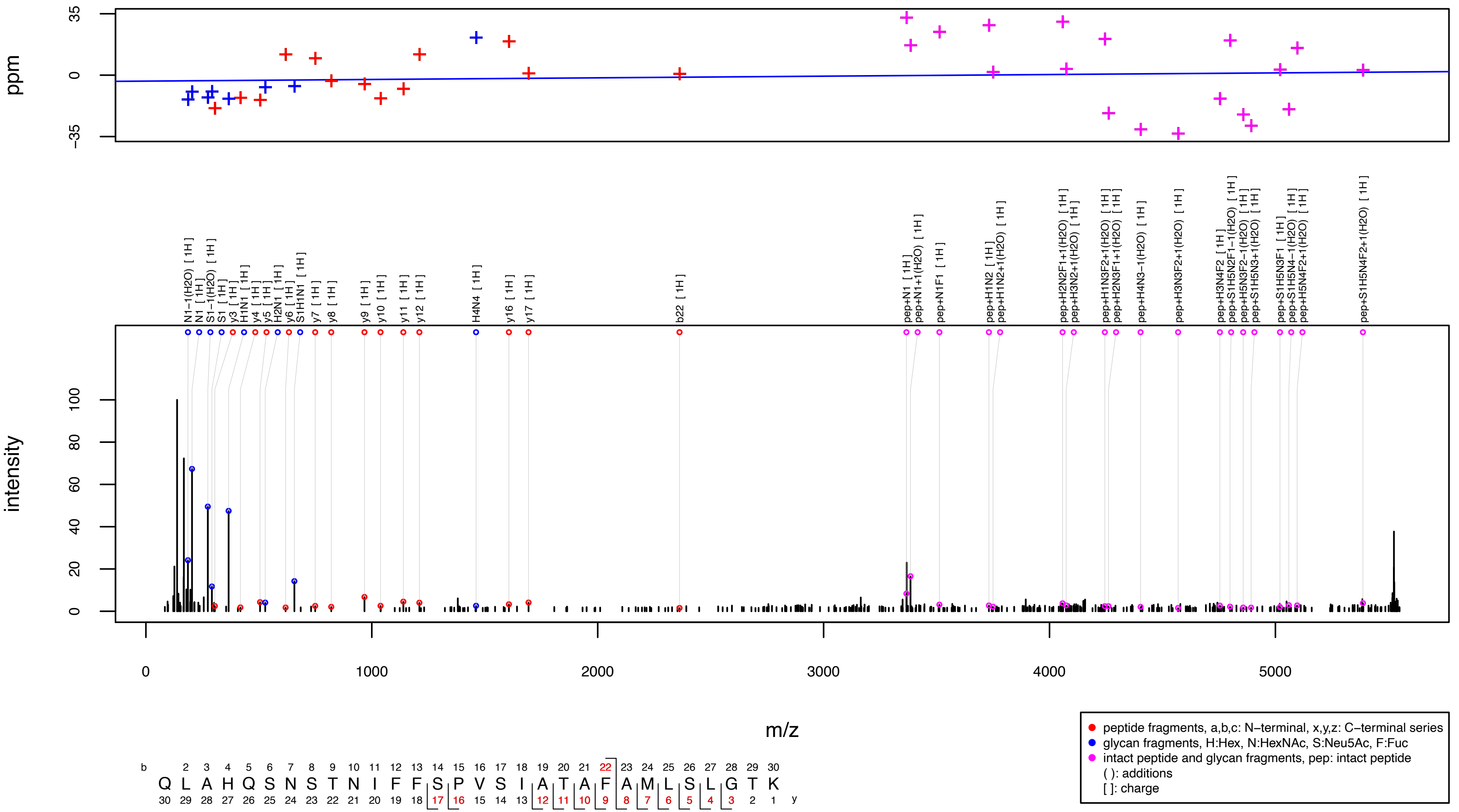

Glycopeptide ID – *Mon Dec 2 18:05:46 2019*

m/z 1273.3286 charge 4 scan 0–0

Score= 107.06 , Hits= 35 , Explained Intensity= 0.28

Peptide: A1AT_HUMAN[268,298]:YLGNATAIFFLPDEGKLQHLENELTHDIITK

Glycan: FHNH(H)HN(F)N, H4N3F2

Charge: 4H

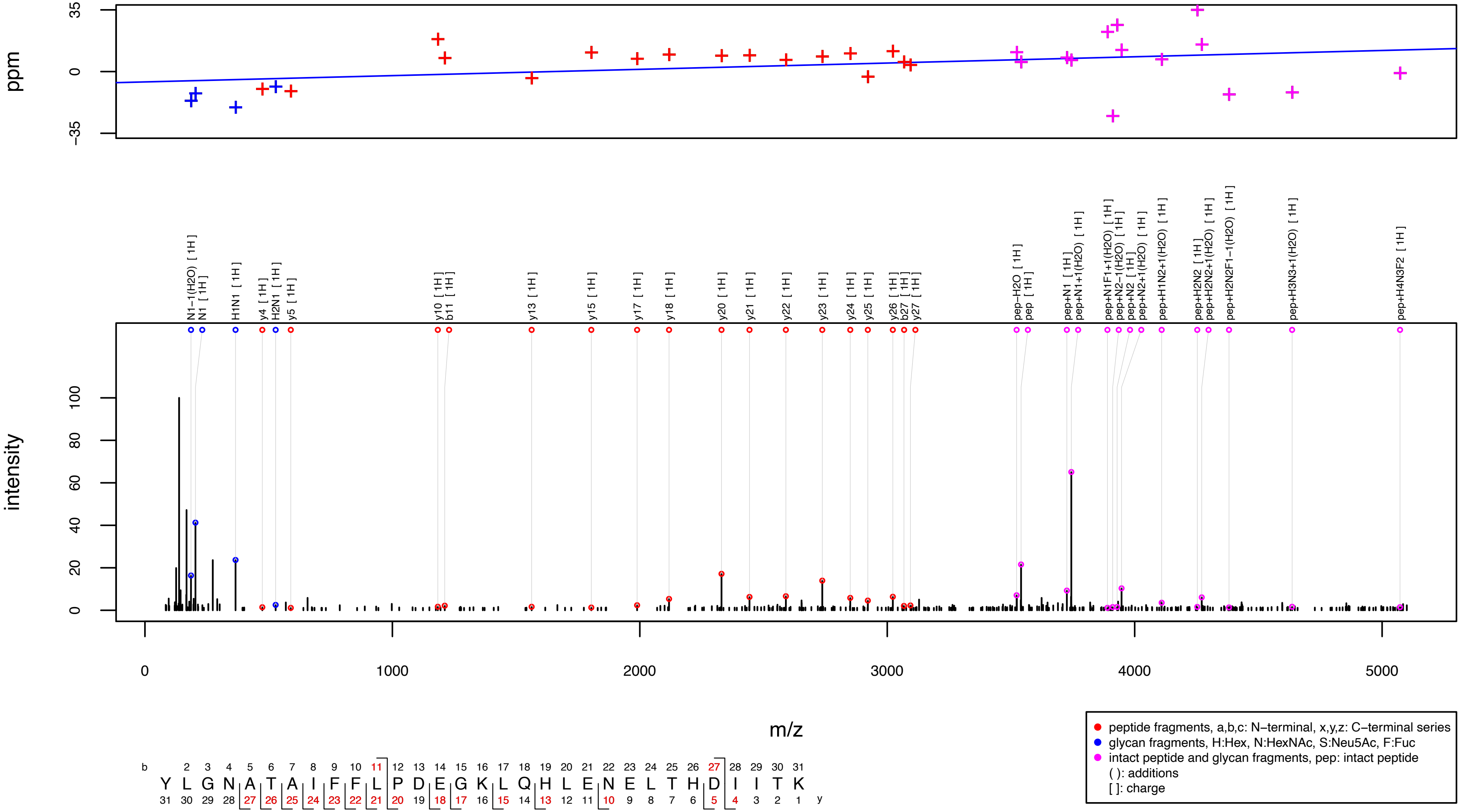

Glycopeptide ID – *Mon Dec 2 18:05:56 2019*

Supplementary Figure 2 GP annotations page 11/27

m/z 1017.0922 charge 5 scan 0–0

Score= 114.45 , Hits= 39 , Explained Intensity= 0.21

Peptide: IGHA1_HUMAN[127,153]:LSLHRPALEDLLLGSEANLTCTLTGLR

Glycan: F(H)NH(F(H)NH)(N)HNN, H5N5F2

Charge: 5H

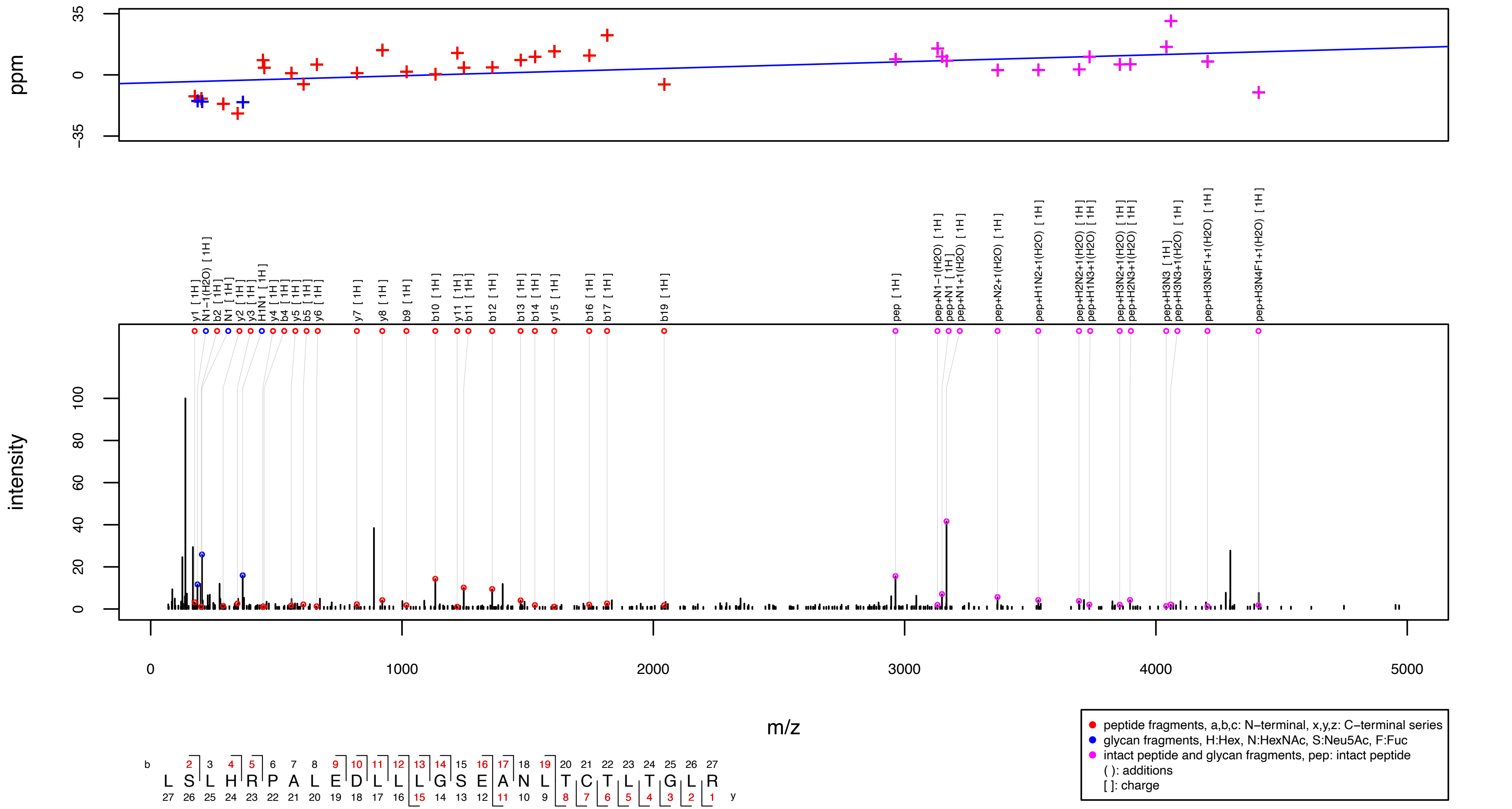

Glycopeptide ID – *Mon Dec 2 18:05:49 2019*

m/z 1230.3131 charge 4 scan 0–0

Score= 166.76 , Hits= 53 , Explained Intensity= 0.46

Peptide: IGHA1_HUMAN[127,153]:LSLHRPALEDLLLGSEANLTCTLTGLR

Glycan: SHNH(NH)(N)HNN, S1H4N5

Charge: 4H

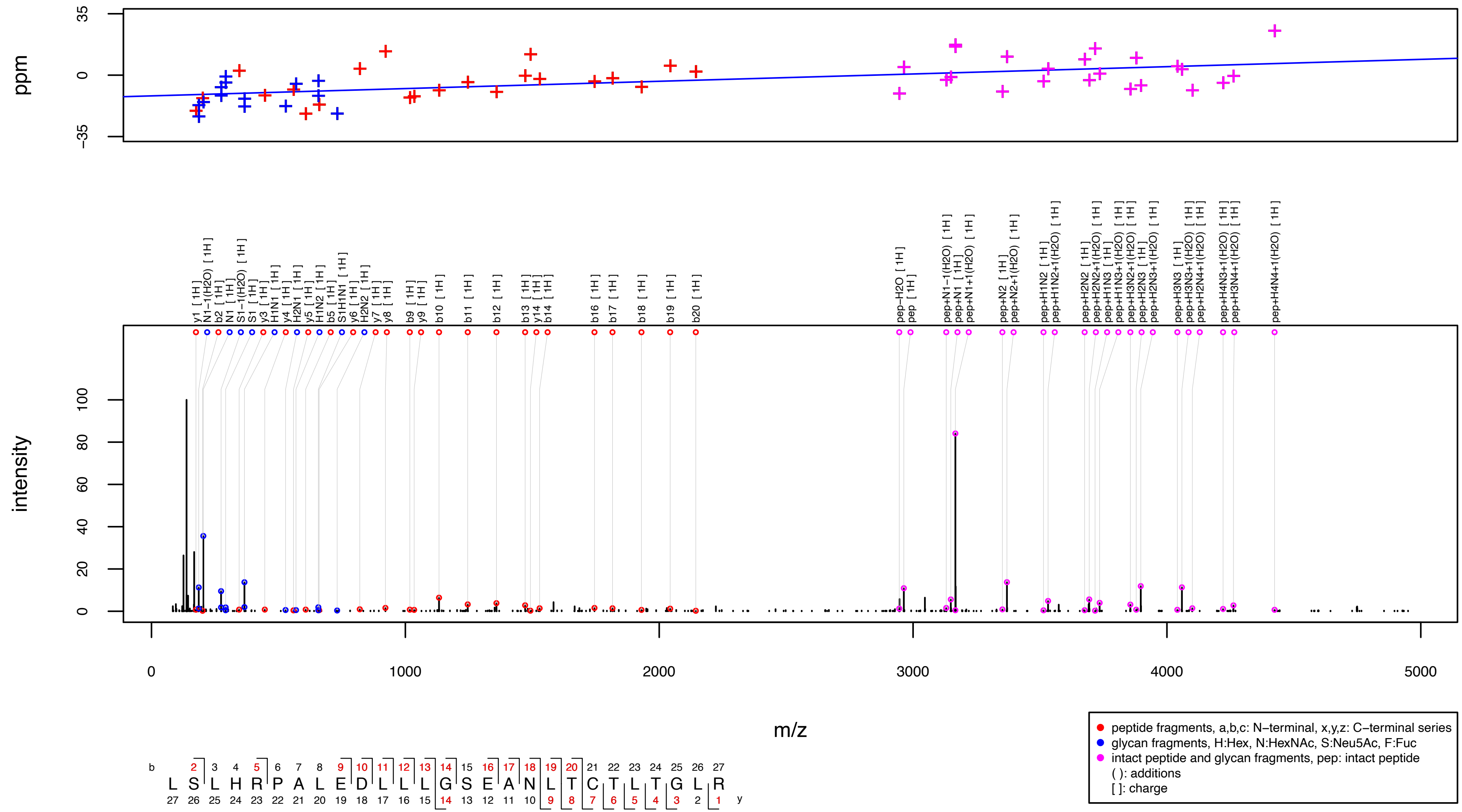

Glycopeptide ID – *Mon Dec 2 18:05:47 2019*

m/z 1060.978 charge 4 scan 0–0

Score= 128.68 , Hits= 42 , Explained Intensity= 0.35

Peptide: HPT_HUMAN[236,253]:VVLHPNYSQVDIGLIKLK

Glycan: SHNH(SHNH)HNN, S2H5N4

Charge: 4H

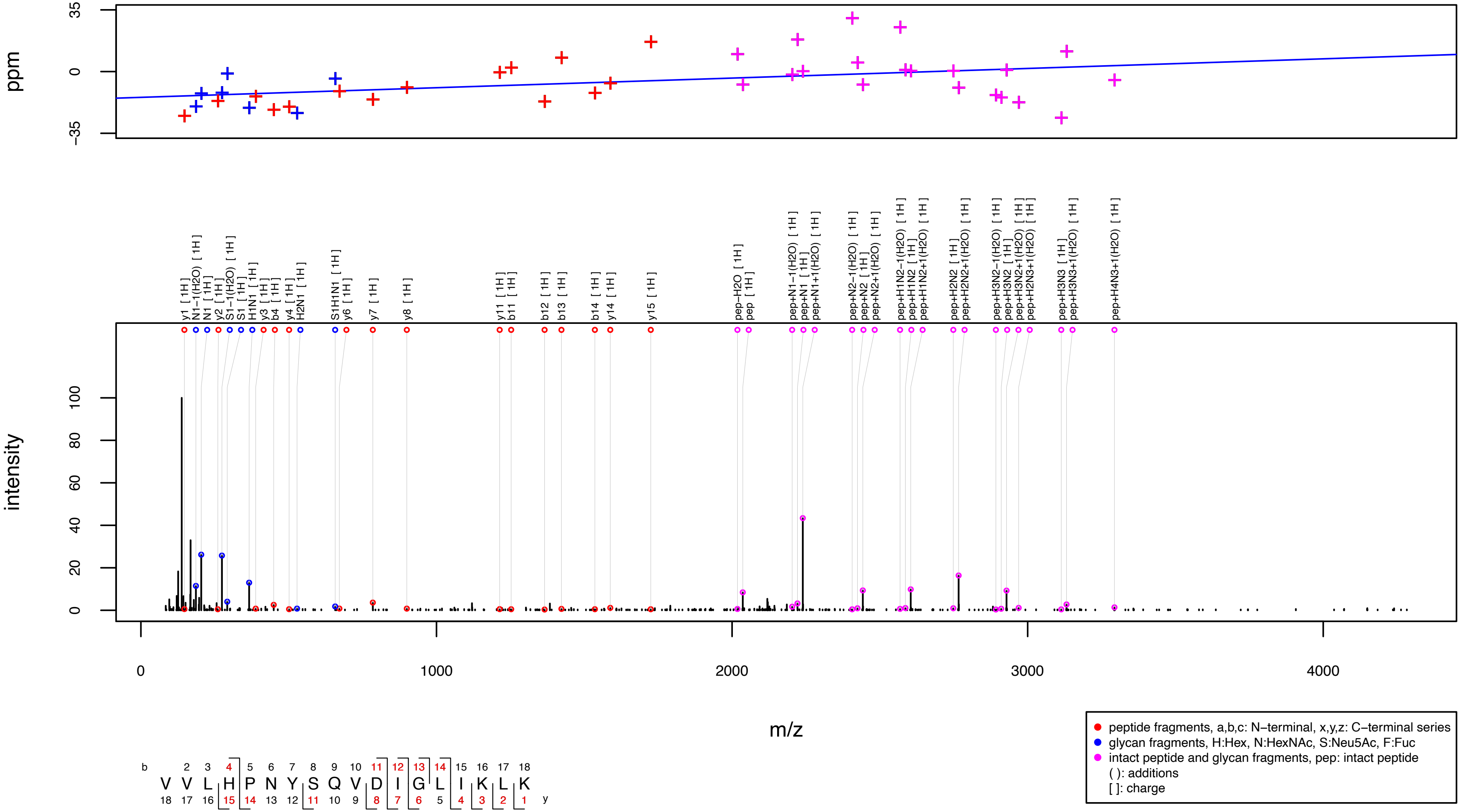

Glycopeptide ID – *Mon Dec 2 18:06:02 2019*

m/z 1216.039 charge 4 scan 0–0

Score= 103.34 , Hits= 36 , Explained Intensity= 0.18

Peptide: IGHA1_HUMAN[127,153]:LSLHRPALEDLLLGSEANLTCTLTGLR

Glycan: SHNH(NH)HN(F)N, S1H4N4F1

Charge: 4H

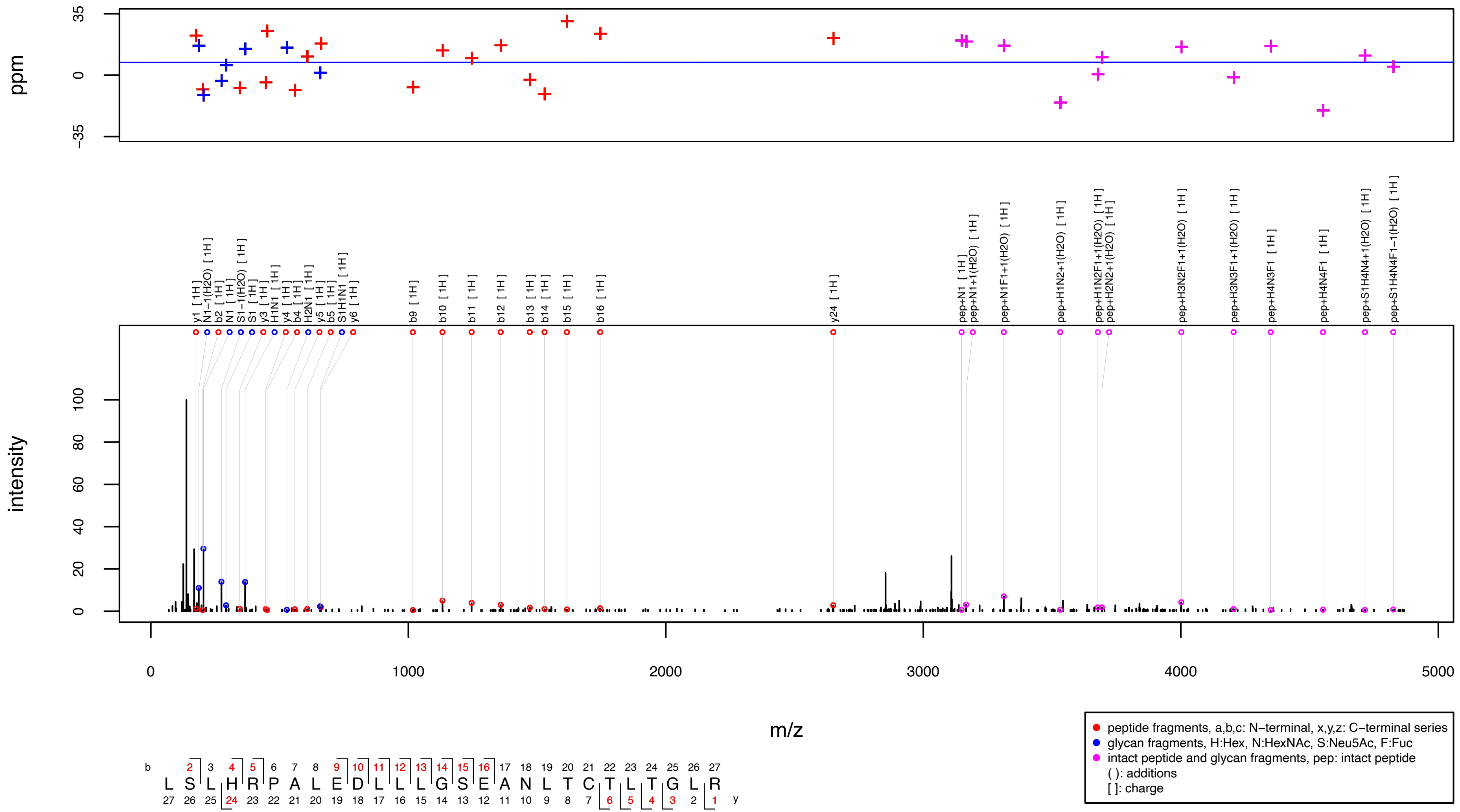

Glycopeptide ID – *Mon Dec 2 18:06:02 2019*

m/z 812.3417 charge 4 scan 0–0

Score= 141.81 , Hits= 45 , Explained Intensity= 0.55

Peptide: IGHG2_HUMAN[168,180]:TKPREEQFNSTFR
Glycan: HNH(NH)HN(F)N, H4N4F1
Charge: 4H

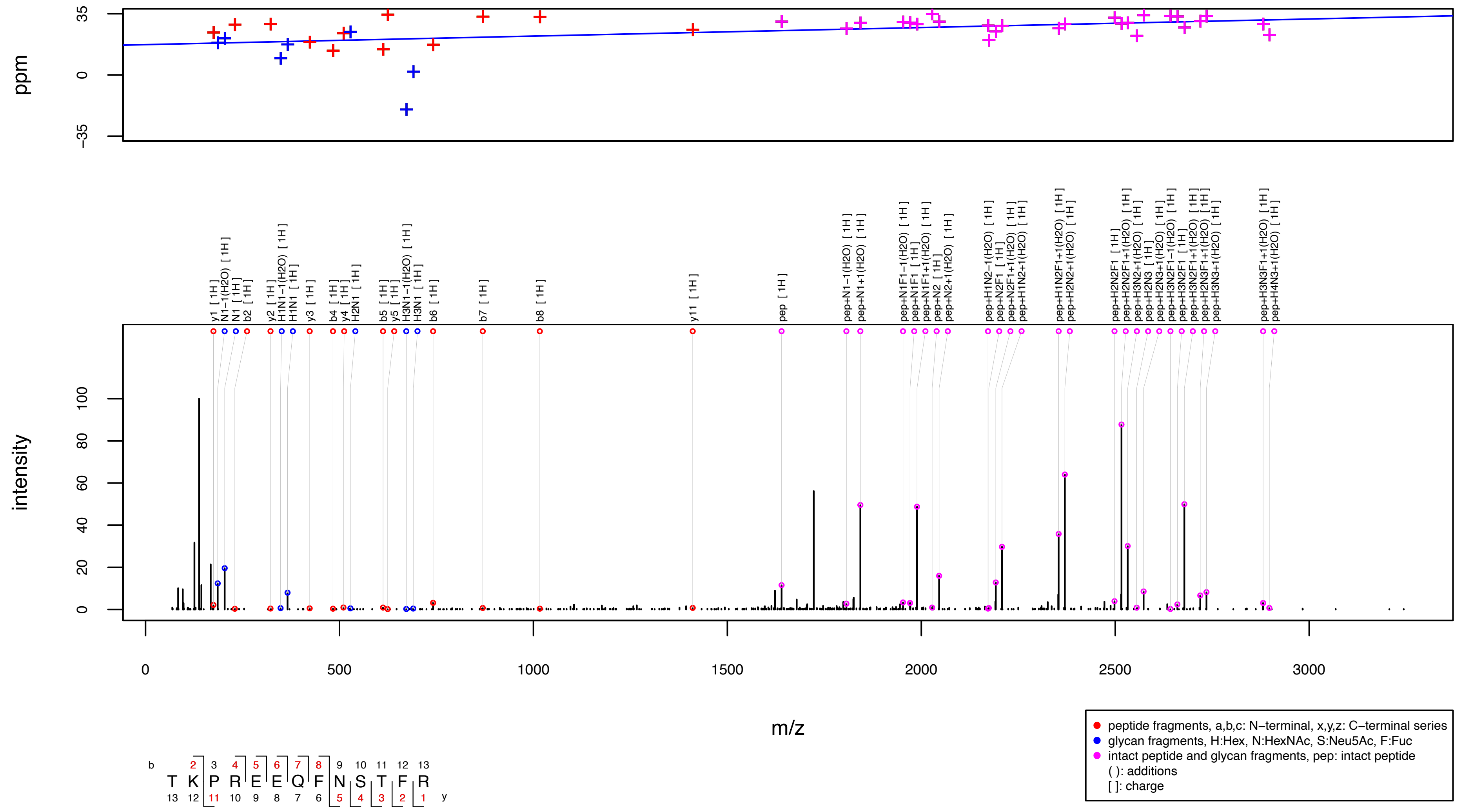

Glycopeptide ID – *Mon Dec 2 18:05:44 2019*

m/z 852.8558 charge 4 scan 0–0

Score= 141.01 , Hits= 44 , Explained Intensity= 0.57

Peptide: IGHG2_HUMAN[168,180]:TKPREEQFNSTFR

Glycan: HNH(HNH)HN(F)N, H5N4F1

Charge: 4H

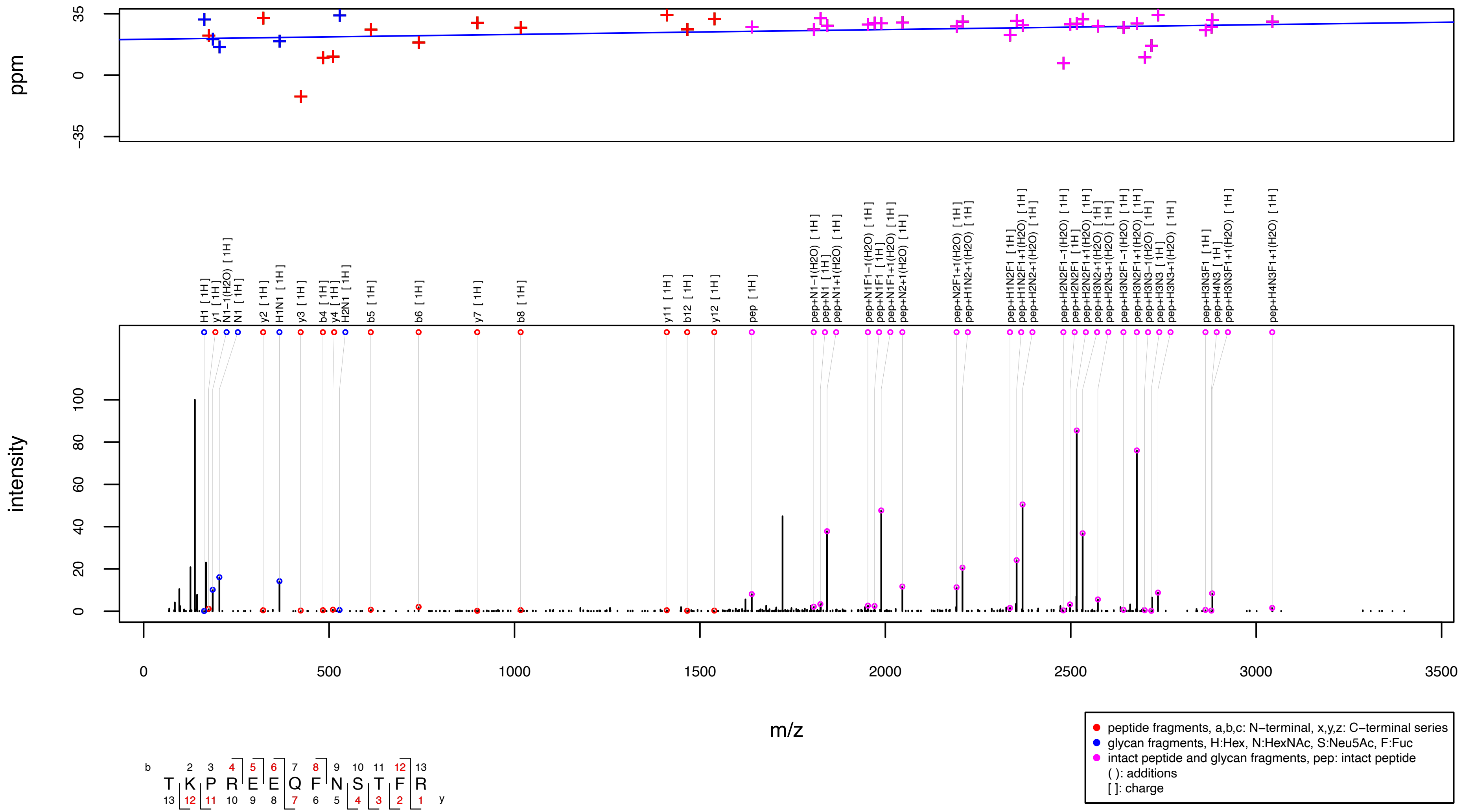

Glycopeptide ID – *Mon Dec 2 18:05:43 2019*

m/z 868.0121 charge 3 scan 0–0

Score= 177.55 , Hits= 57 , Explained Intensity= 0.46

Peptide: IGHG2_HUMAN[172,180]:EEQFNSTFR
Glycan: NNH(H)HN(F)N, H3N4F1
Charge: 3H

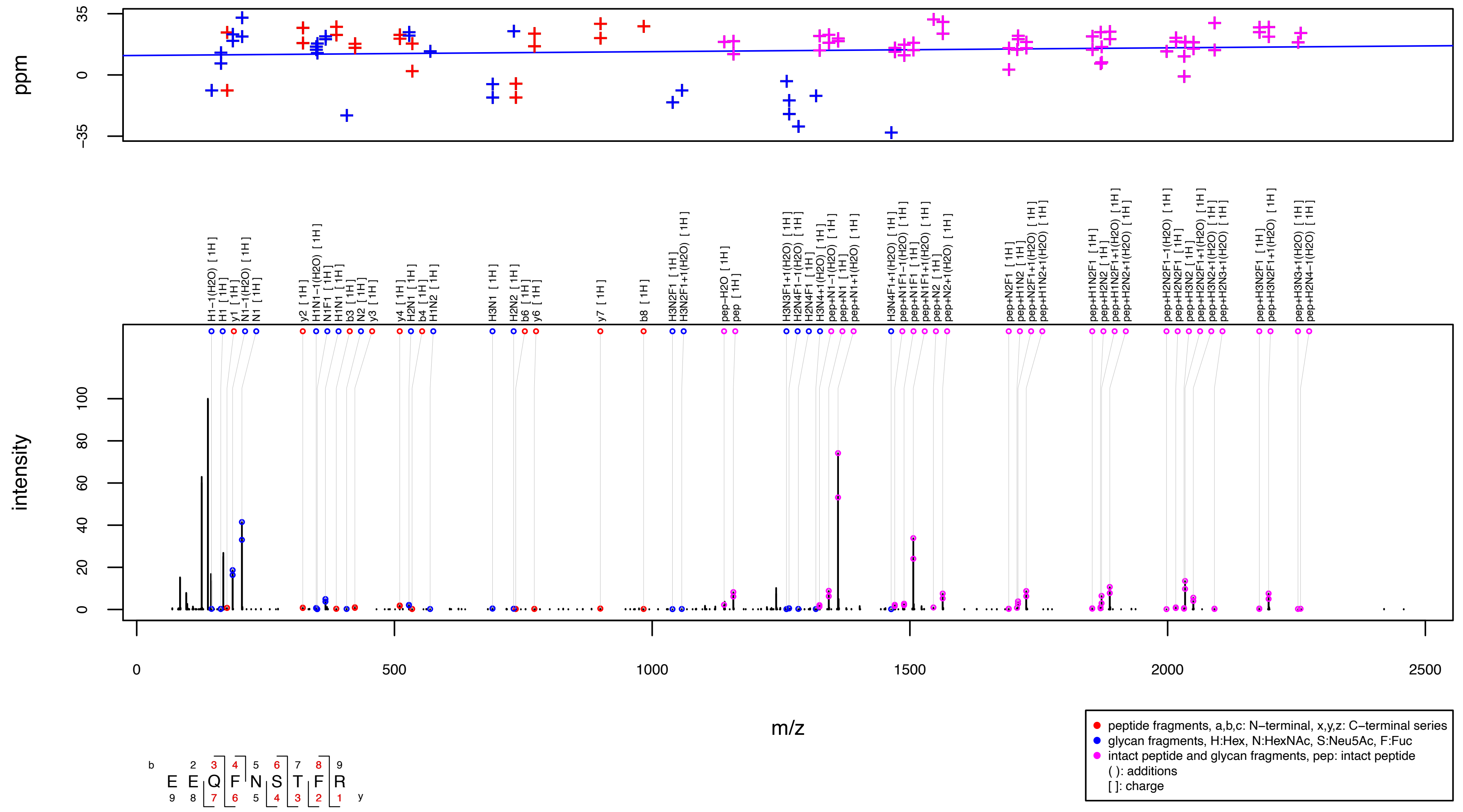

Supplementary Figure 2 GP annotations page 18/27

m/z 922.0283 charge 3 scan 0–0

Score= 148.42 , Hits= 46 , Explained Intensity= 0.51

Peptide: IGHG2_HUMAN[172,180]:EEQFNSTFR

Glycan: HNH(NH)HN(F)N, H4N4F1

Charge: 3H

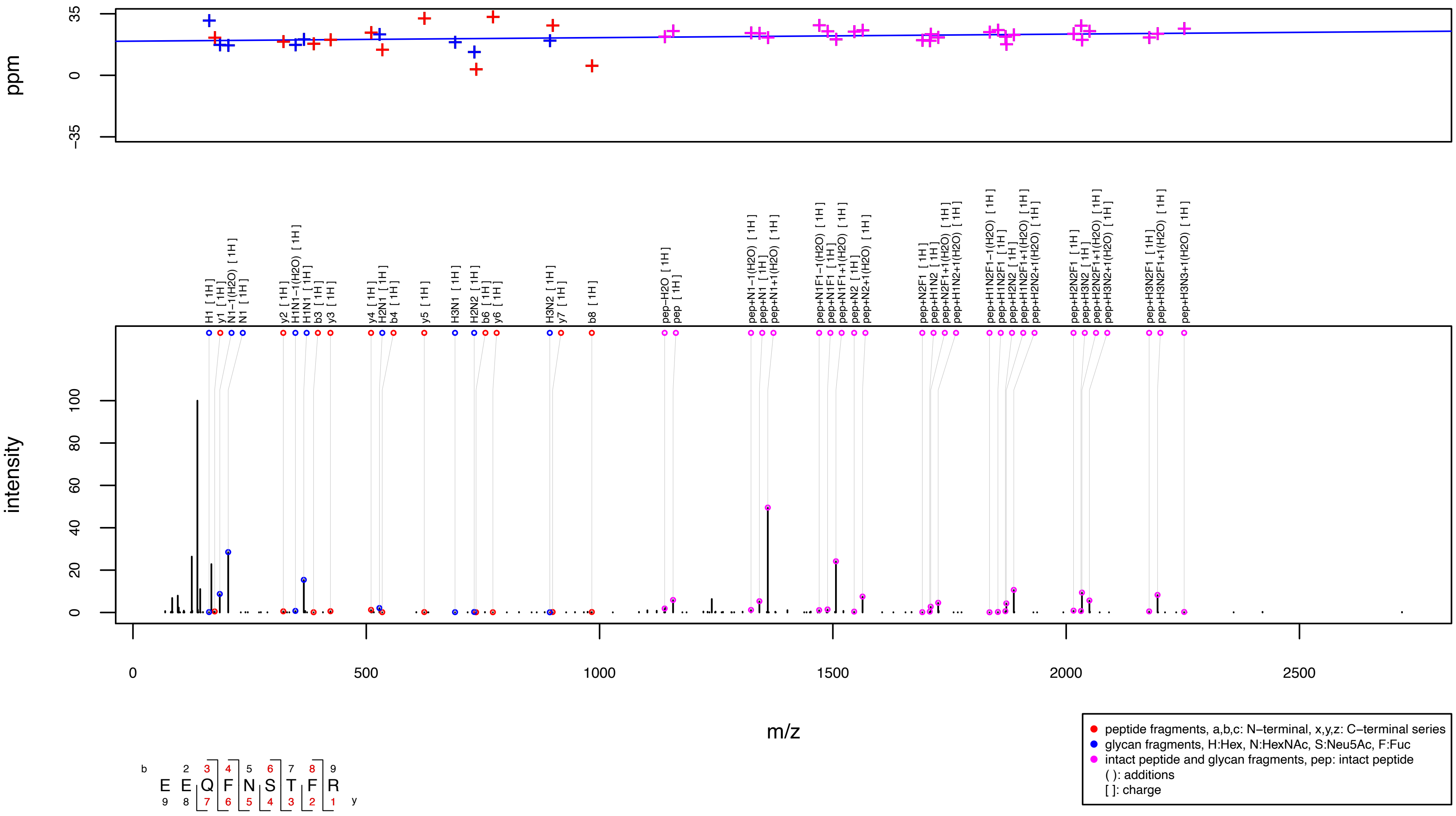

Glycopeptide ID – *Mon Dec 2 18:06:03 2019*

m/z 1347.338 charge 4 scan 0–0

Score= 192.86 , Hits= 59 , Explained Intensity= 0.53

Peptide: A1AT_HUMAN[64,93]:QLAHQSNSTNIFFSPVSIATAFAMLSLGTK

Glycan: SHNH(SHNH)HNN, S2H5N4

Charge: 4H

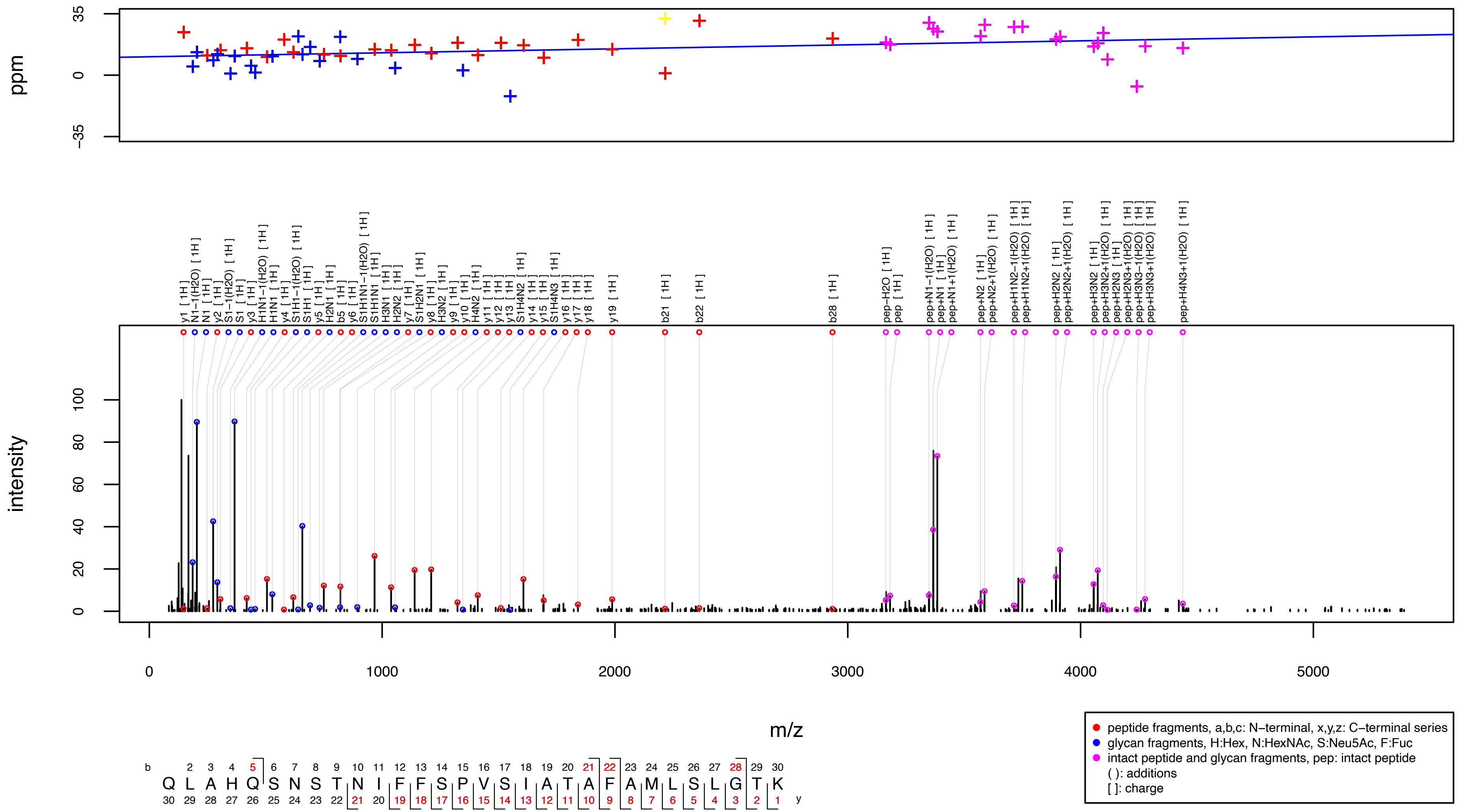

Glycopeptide ID – *Mon Dec 2 18:05:46 2019*

Supplementary Figure 2 GP annotations page 20/27

m/z 976.2347 charge 5 scan 0–0

Score= 133.61 , Hits= 41 , Explained Intensity= 0.46

Peptide: IGHA1_HUMAN[127,153]:LSLHRPALEDLLLGSEANLTCTLTGLR
Glycan: SHNH(HNH)HNN, S1H5N4
Charge: 5H

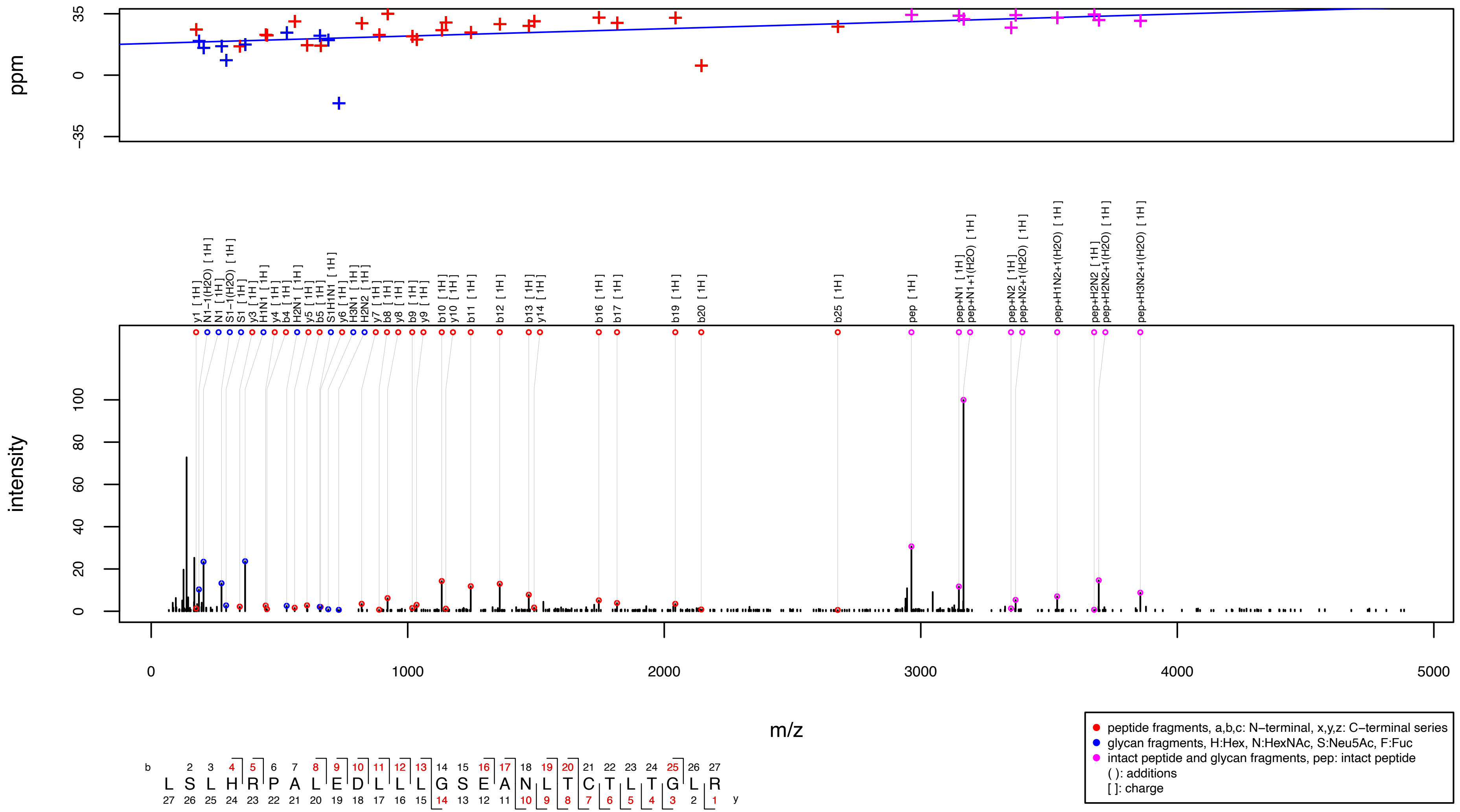

Glycopeptide ID – *Mon Dec 2 18:05:48 2019*

m/z 1270.8234 charge 4 scan 0–0

Score= 157.48 , Hits= 50 , Explained Intensity= 0.58

Peptide: IGHA1_HUMAN[127,153]:LSLHRPALEDLLLGSEANLTCTLTGLR

Glycan: SHNH(HNH)(N)HNN, S1H5N5

Charge: 4H

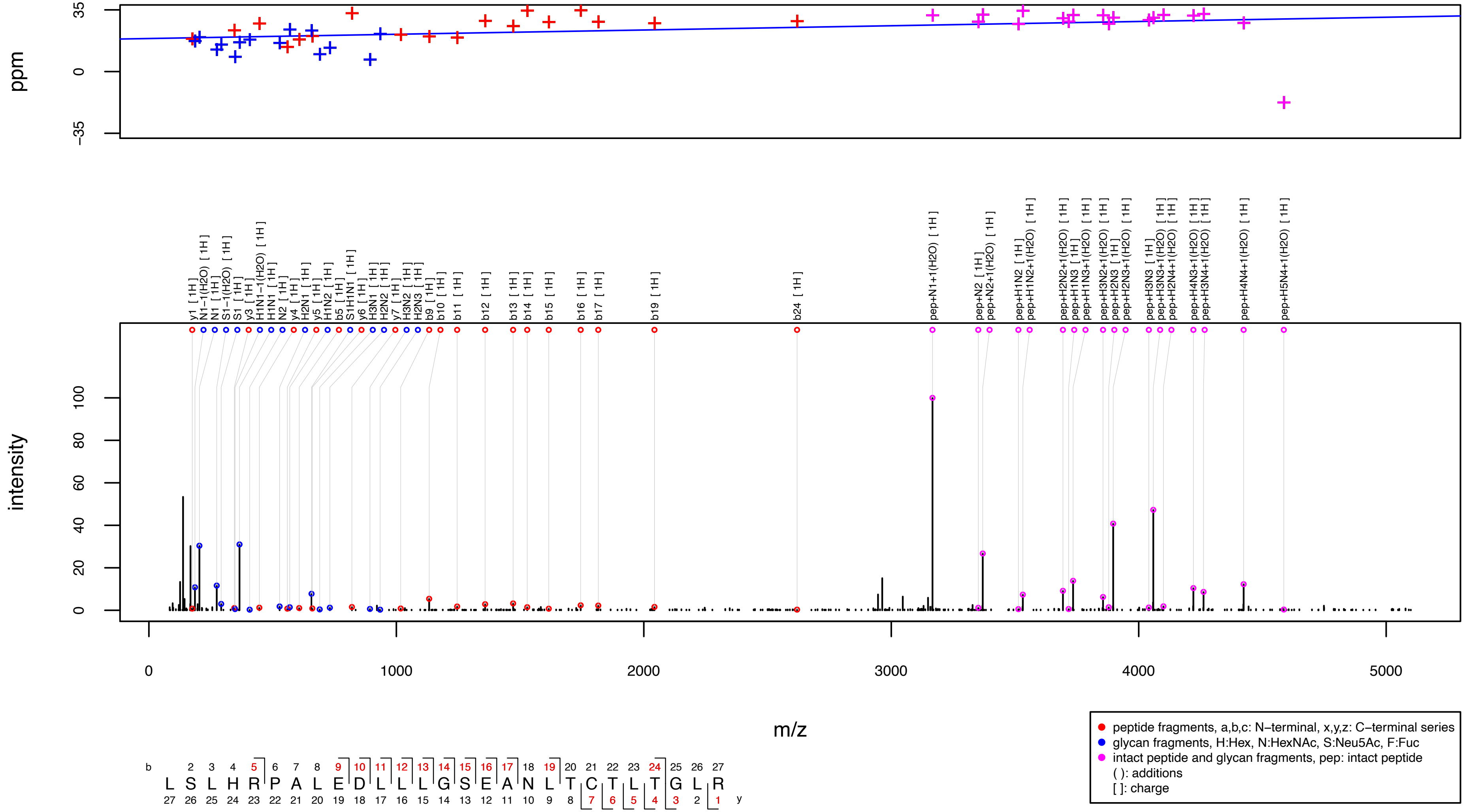

Glycopeptide ID – *Mon Dec 2 18:05:58 2019*

Supplementary Figure 2 GP annotations page 22/27

m/z 1000.6844 charge 4 scan 0–0

Score= 188.81 , Hits= 58 , Explained Intensity= 0.55

Peptide: HPT_HUMAN[236,251]:VVLHPNYSQVDIGLIK

Glycan: SHNH(SHNH)HNN, S2H5N4

Charge: 4H

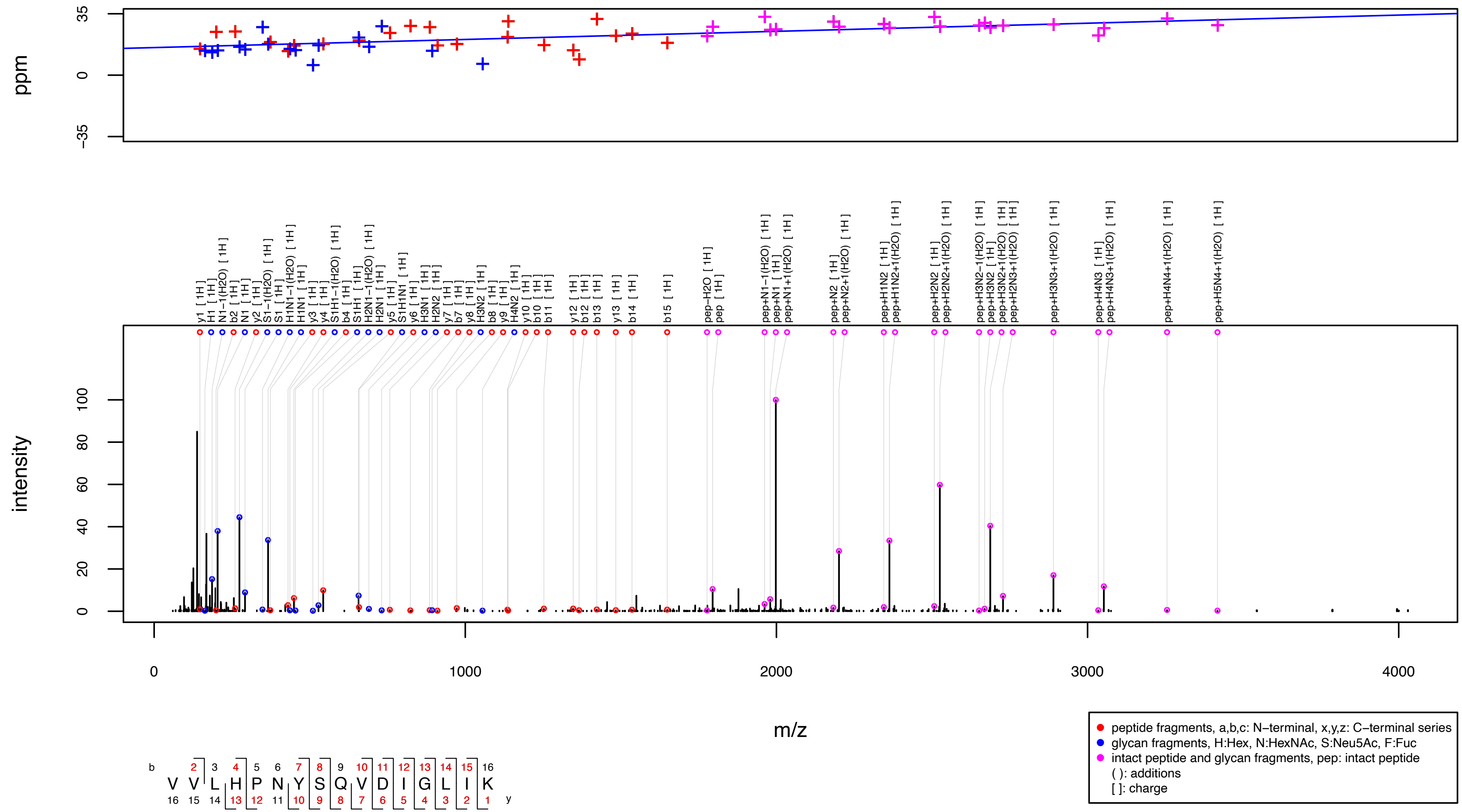

Glycopeptide ID – *Mon Dec 2 18:05:45 2019*

Supplementary Figure 2 GP annotations page 23/27

m/z 1220.0497 charge 4 scan 0–0

Score= 164.74 , Hits= 51 , Explained Intensity= 0.58

Peptide: IGHA1_HUMAN[127,153]:LSLHRPALEDLLLGSEANLTCTLTGLR

Glycan: SHNH(HNH)HNN, S1H5N4

Charge: 4H

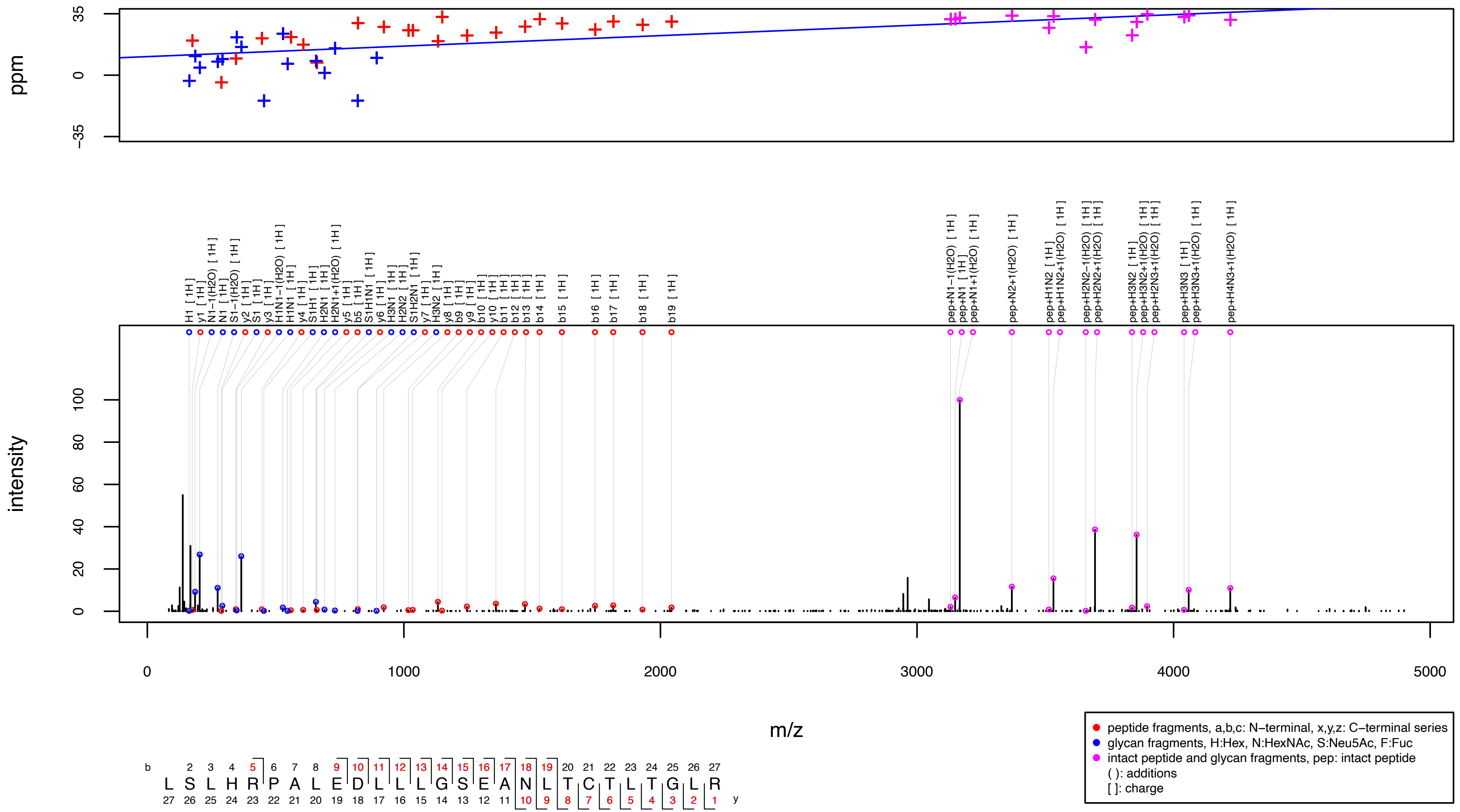

Glycopeptide ID – *Mon Dec 2 18:05:51 2019*

m/z 984.4412 charge 5 scan 0–0

Score= 105.31 , Hits= 35 , Explained Intensity= 0.31
Peptide: IGHA1_HUMAN[127,153]:LSLHRPALEDLLLGSEANLTCTLTGLR
Glycan: SHNH(NH)(N)HNN, S1H4N5
Charge: 5H

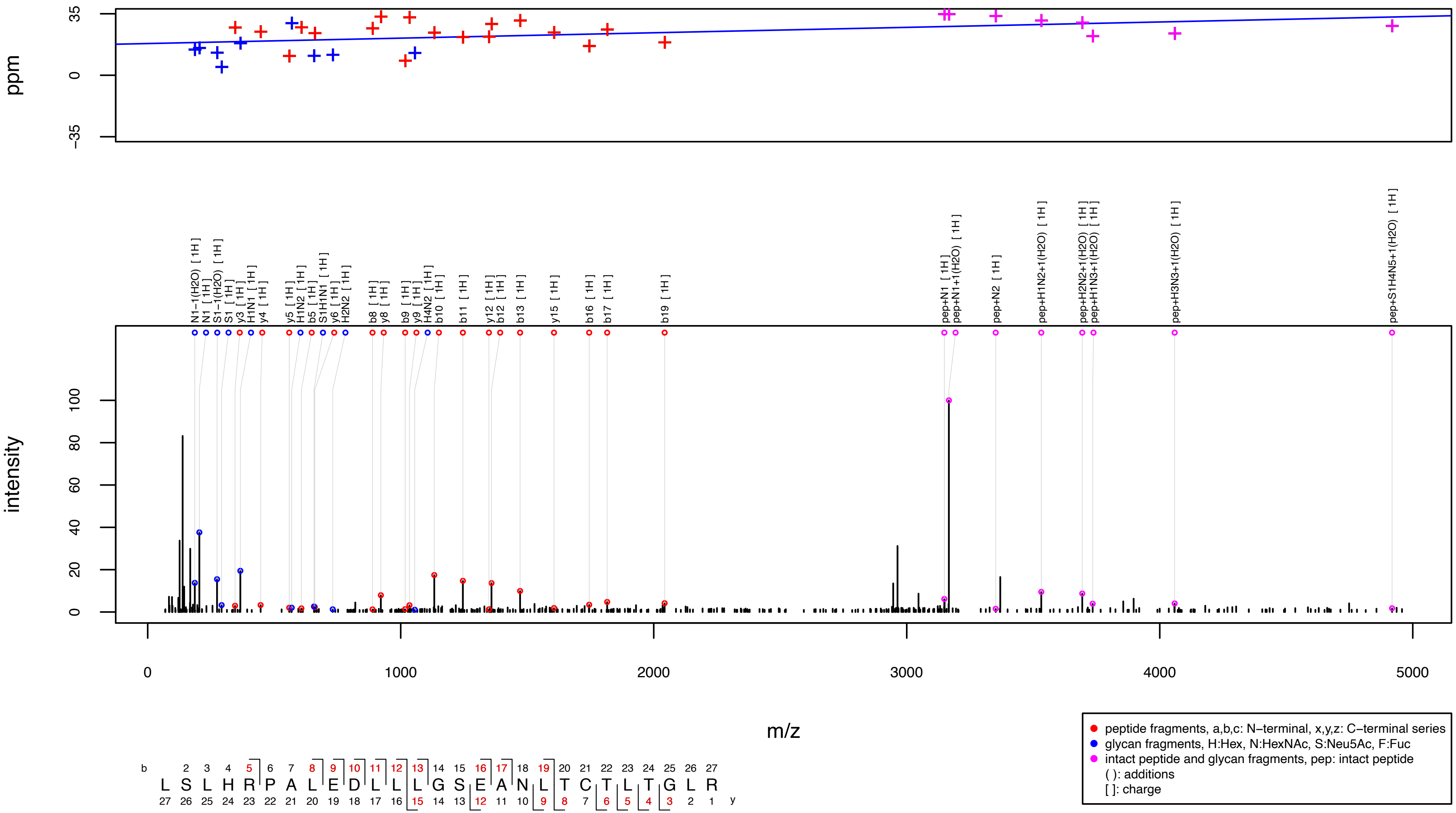

Glycopeptide ID – *Mon Dec 2 18:05:59 2019*

Supplementary Figure 2 GP annotations page 25/27

m/z 1078.0559 charge 5 scan 0–0

Score= 158.27 , Hits= 49 , Explained Intensity= 0.41

Peptide: A1AT_HUMAN[64,93]:QLAHQSNSTNIFFSPVSIATAFAMLSLGTK

Glycan: SHNH(SHNH)HNN, S2H5N4

Charge: 5H

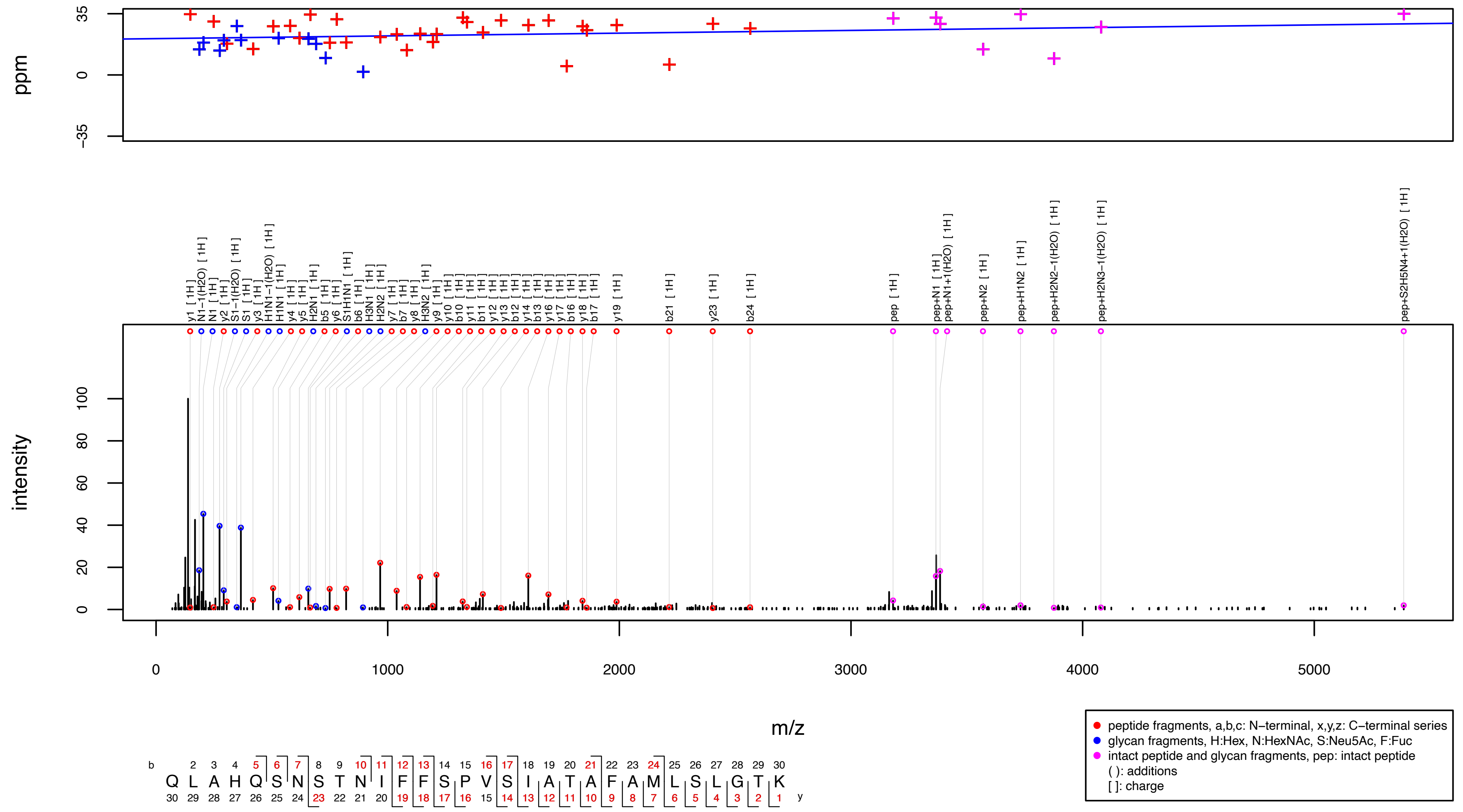

Glycopeptide ID – *Mon Dec 2 18:05:42 2019*

m/z 1117.0226 charge 4 scan 0–0

Score= 175.48 , Hits= 54 , Explained Intensity= 0.61

Peptide: IGHA1_HUMAN[127,153]:LSLHRPALEDLLLGSEANLTCTLTGLR

Glycan: NH(NH)(N)HNN, H3N5

Charge: 4H

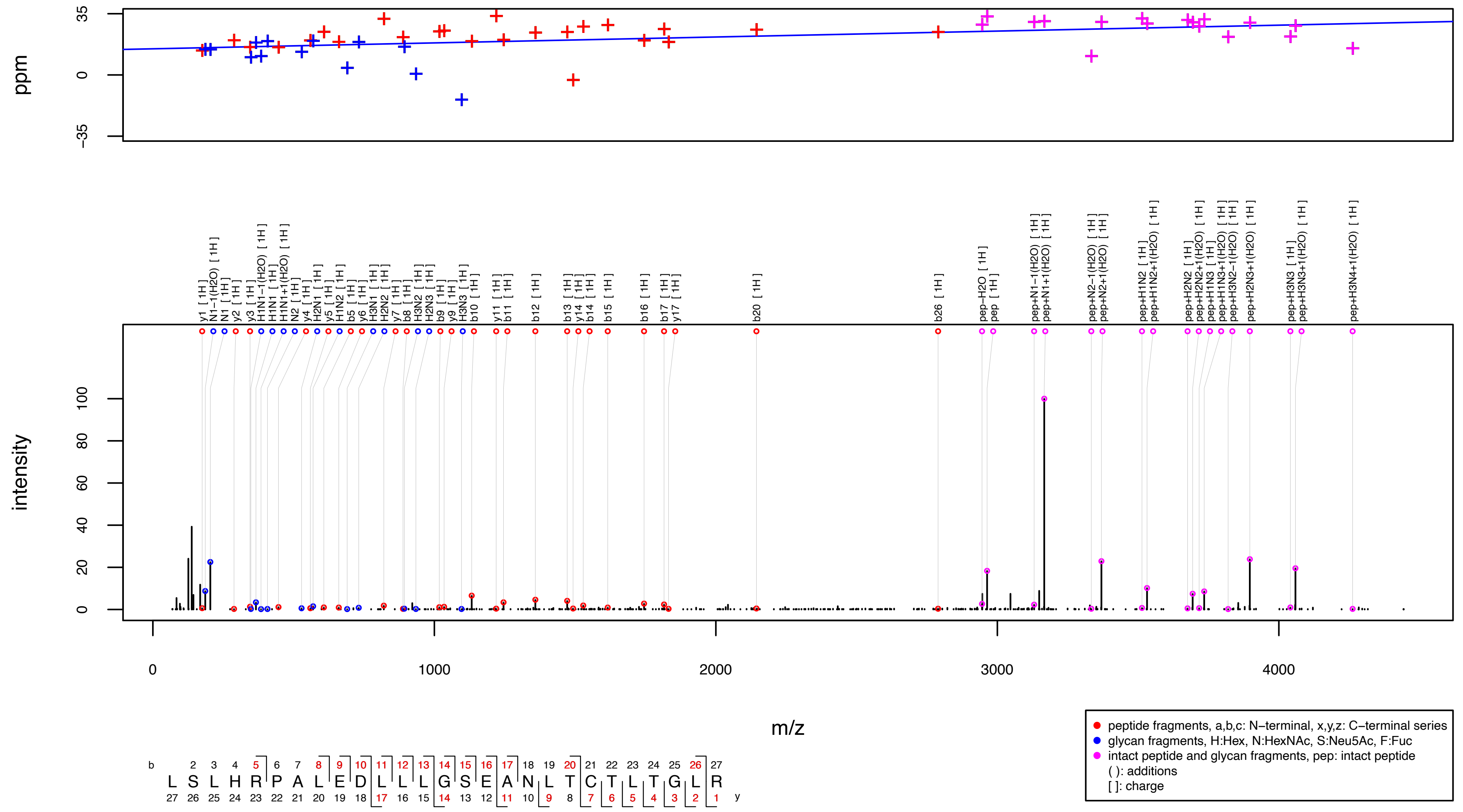

Glycopeptide ID – *Mon Dec 2 18:05:45 2019*
